# Comparison of FcRn- and pIgR-Mediated Transport in MDCK Cells by Fluorescence Confocal Microscopy

**DOI:** 10.1111/j.1600-0854.2010.01083.x

**Published:** 2010-06-29

**Authors:** Galina V Jerdeva, Devin B Tesar, Kathryn E Huey-Tubman, Mark S Ladinsky, Scott E Fraser, Pamela J Bjorkman

**Affiliations:** 1 Division of Biology, California Institute of Technology, 1200 East California BoulevardPasadena, CA 91125, USA; 2 Howard Hughes Medical Institute, California Institute of Technology, 1200 East California BoulevardPasadena, CA 91125, USA

**Keywords:** apical, basolateral, FcRn, live-cell imaging, Madin–Darby canine kidney (MDCK) cells, pIgR, pulse-chase, transcytosis

## Abstract

Protein delivery across polarized epithelia is controlled by receptor-mediated transcytosis. Many studies have examined basolateral-to-apical trafficking of polymeric IgA (pIgA) by the polymeric immunoglobulin receptor (pIgR). Less is known about apical-to-basolateral transcytosis, the direction the neonatal Fc receptor (FcRn) transports maternal IgGs across intestinal epithelia. To compare apical-to-basolateral and basolateral-to-apical transcytosis, we co-expressed FcRn and pIgR in Madin-Darby canine kidney (MDCK) cells and used pulse-chase experiments with confocal microscopy to examine transport of apically applied IgG Fcγ and basolaterally applied pIgA. Fcγ and pIgA trafficking routes were initially separate but intermixed at later chase times. Fcγ was first localized near the apical surface, but became more equally distributed across the cell, consistent with concomitant transcytosis and recycling. By contrast, pIgA transport was strongly unidirectional: pIgA shifted from near the basolateral surface to an apical location with increasing time. Some Fcγ and pIgA fluorescence colocalized in early (EEA1-positive), recycling (Rab11a-positive), and transferrin (Tf)-positive common/basolateral recycling endosomes. Fcγ became more enriched in Tf-positive endosomes with time, whereas pIgA was sorted from these compartments. Live-cell imaging revealed that vesicles containing Fcγ or pIgA shared similar mobility characteristics and were equivalently affected by depolymerizing microtubules, indicating that both trafficking routes depended to roughly the same extent on intact microtubules.

Epithelia represent a fundamental type of multi-cellular organization, lining numerous internal and external surfaces of the body and forming a selective barrier between the lumen of an organ and the underlying tissue. The apical and basolateral domains of polarized epithelia maintain distinct protein and lipid compositions (1). Paracellular transport or transport of macromolecules between epithelial cells in polarized cell monolayers is usually prevented by tight junctions (2). Most proteins that cross polarized epithelial cell barriers do so via receptor-mediated transcytosis. This mode of protein transport consists of three main steps: endocytosis (internalization) from the apical or basolateral side of the cell, transport of vesicles containing receptor–ligand complexes across the cell, and exocytosis (release) of ligand at the opposite cell surface (reviewed in 3).

Although a variety of receptors have been studied, the transport pathways of only a few have been characterized in detail. One such receptor, the polymeric immunoglobulin receptor (pIgR), transports polymeric immunoglobulins across mucosal epithelia into mucosal secretions. The receptor is expressed on the basolateral surface of epithelial cells, where it binds to polymeric immunoglobulins (such as polymeric IgA; pIgA) secreted by local plasma cells (4–6). pIgR–pIgA complexes are internalized from the basolateral surface via clathrin-coated pits and delivered to basolateral early endosomes (7). Cargo that is taken up by non-receptor-mediated fluid phase endocytosis is believed to be sorted to late endosomes and lysosomes, while the majority of pIgR–pIgA complexes, together with transferrin (Tf) and its receptor, are routed to common recycling endosomes in the subapical region. After exit from the common recycling endosomes, pIgR–pIgA is transported to apical recycling endosomes and delivered to the apical cell surface where pIgR is proteolytically cleaved, releasing the pIgR ectodomain (secretory component) covalently bound to pIgA (now secretory IgA) into secretions (8–12). The fraction of pIgR–pIgA that escapes cleavage is directed to apical early and recycling endosomes for recycling back to the apical surface to ensure eventual cleavage of all complexes (13–15). The cleavage of pIgR at the apical surface assures that it functions primarily in basolateral-to-apical transcytosis, although uncleaved pIgR can endocytose pIgA from the apical surface, eventually recycling it back to the same surface (16).

Less is known about receptors that transport ligands in the apical-to-basolateral direction. One such receptor is the neonatal Fc receptor (FcRn), a class I MHC-related protein that transports maternal IgG across epithelial cell barriers to provide immunity to fetal or newborn mammals (17,18). FcRn is expressed at the apical surface of the polarized epithelium of the intestine of newborn suckling rodents, where it binds to IgG from ingested milk at acidic pH, transports it across the epithelium in acidic intracellular vesicles, and then releases it into the neonatal bloodstream when FcRn–IgG complexes are exposed to the slightly basic pH of the blood (approximately pH 7.4) (17). A sharply pH-dependent binding to IgG is crucial for FcRn function; thus, FcRn binds to IgG with a nanomolar affinity at pH 6.5 and below but shows no detectable binding to IgG at neutral or basic pH (17,19–21).

Although FcRn was first discovered in neonates, the receptor also functions in adult mammals to extend the serum half-life of IgG by protecting it from a default degradative pathway in vascular endothelial cells (22–24) and hematopoetic cells (25,26). In its role as a protection receptor, the pH-dependent binding permits FcRn to bind tightly to IgG in acidic vesicles, thereby preventing its release in degradative compartments and facilitating recycling back to the cell surface, where it is released upon encountering the slightly basic pH of the bloodstream. In addition, FcRn engages in bidirectional transcytosis across epithelial cell barriers. For example, FcRn transfers IgG via basolateral-to-apical transport into luminal secretions of the adult human gastrointestinal system (27), where the IgG scavenges luminal antigens for recognition by the immune system, and then transcytoses the IgG–antigen complexes back across the epithelium (apical-to-basolateral transport) into the lamina propria for processing by dendritic cells and presentation to CD4+ T cells (28). FcRn is also present in adult human lungs (29–31), where it has been shown to deliver inhaled IgG and Fcγ fusion proteins into the bloodstream, suggesting a function for FcRn-mediated transcytosis in the lung that is currently being exploited for delivery of protein drugs (29,30,32).

Much of what is known about transcytotic pathways comes from studies of pIgR-mediated transcytosis of pIgA in Madin–Darby canine kidney (MDCK) cells (12,33–35). This cultured cell line forms a well-polarized epithelial monolayer when grown on permeable filters (1). pIgR expressed from cloned cDNA functions in these cells to deliver pIgA from the basolateral side of the cell to the apical surface as *in vivo*(36). MDCK cells have also been used for studying transcytosis and recycling of IgG by FcRn (37–42), revealing species-specific differences between human FcRn and rat FcRn: human FcRn transcytoses IgG predominantly in the basolateral-to-apical direction (39), whereas rat FcRn transports IgG predominantly in the apical-to-basolateral direction in MDCK cells (40) and IMCD cells (43). While quantitative studies such as these have yielded insight into the preferred directionality and kinetics of FcRn-mediated transcytosis, the mechanistic features of this process remain poorly understood. Light-microscopy studies of FcRn trafficking over the past decade have largely focused on FcRn-mediated recycling in cultured endothelial cells (44–48). Only a limited number of recent studies have begun to elucidate mechanistic aspects of FcRn-mediated transcytosis using RNA interference technology (42) and electron tomography (49).

Here we used quantitative fluorescence confocal imaging to compare pIgR- and FcRn-mediated transcytosis in MDCK cells. By expressing human pIgR and rat FcRn in the same cells, we were able to observe trafficking of these receptors as their ligands were transported in opposite directions (basolateral-to-apical for pIgR transcytosis of pIgA; apical-to-basolateral for FcRn transcytosis of Fcγ), and in separate experiments, when they were transported in the same direction. We combined this same-cell comparison of apical-to-basolateral and basolateral-to-apical transcytosis (performed for the first time to our knowledge) with kinetic analyses using pulse-chase methods, and performed a quantitative comparison of the velocities of transcytotic vesicles in normal and microtubule-disrupted cells with non-transcytotic vesicles (vesicles heading for degradative compartments). We show distinct sorting steps involved in FcRn- and pIgR-dependent trafficking, including sorting through early endosomes, recycling endosomes, and Tf-positive common and basolateral recycling endosomes. Overlaps in the pathways taken by labeled Fcγ and pIgA after they were internalized from the opposite or the same surfaces indicated significant intersections of the two pathways. By analyzing the cellular distribution of these ligand–receptor complexes with respect to three distinct sub-volumes within the cell (apical, medial and basolateral), our results facilitate a detailed analysis of the spatio-temporal distributions of these receptors, both independently and with respect to one another, over the course of their transcytotic itineraries.

## Results and Discussion

### Fcγ and pIgA transit through the same vesicles during late trafficking steps

In order to investigate FcRn-mediated transport of IgG, we used MDCK cells stably expressing rat FcRn (FcRn-MDCK). This cell line specifically transcytoses IgG and Fcγ across polarized cell monolayers (40), offering an *in vitro* system that mimics the *in vivo* FcRn-dependent transport system. Our previous studies using confocal microscopy identified the intracellular compartments involved in transcytosis of labeled Fcγ in FcRn-MDCK cells by colocalization with organelle-specific markers (40). Here we extended these studies to compare the transcytosis mediated by FcRn, a bidirectionally transcytosing receptor, with the trafficking of pIgR, a unidirectional, basolateral-to-apical transcytosing receptor. FcRn-MDCK or untransfected MDCK II cells were infected with a recombinant lentivirus to direct expression of human pIgR. Cells were stained with an anti-pIgR antibody to verify expression of pIgR, which usually ranged from 20% to 40% of cells in any given experiment (data not shown). Double-positive cells (FcRn-pIgR-MDCK) were used to directly compare FcRn/Fcγ and pIgR/pIgA trafficking, and single-positive cells (either FcRn-MDCK or pIgR-MDCK) were used when expression of both receptors was not required.

**Figure 1:**
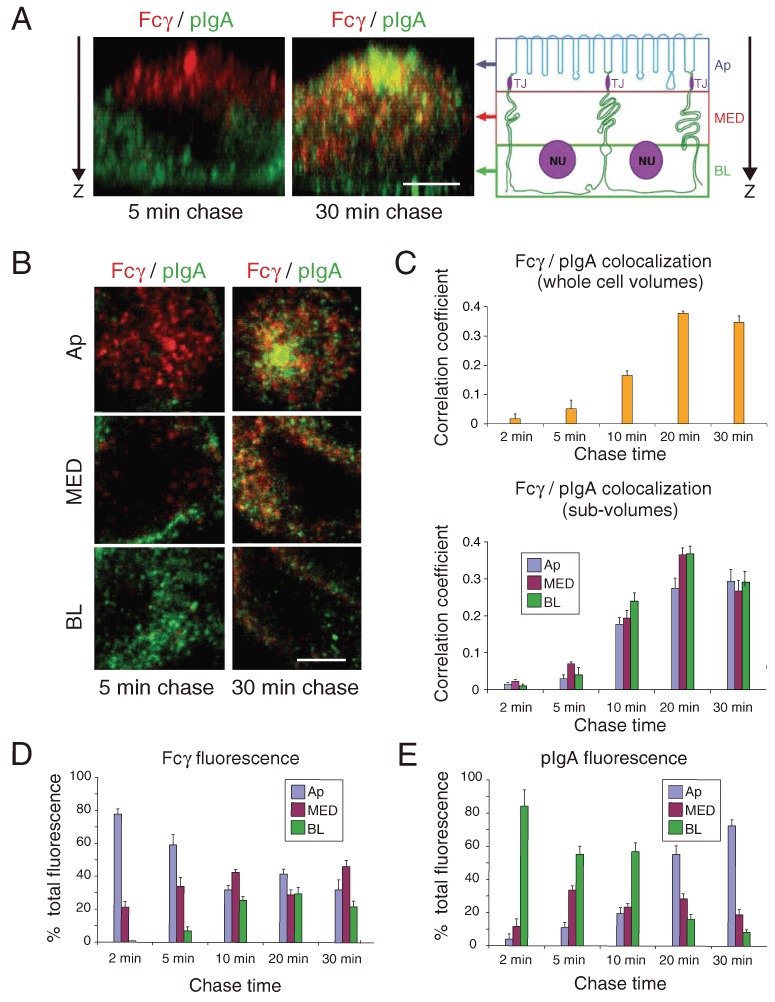
**Colocalization of Fcγ and pIgA.** AlexaFluor-568-labeled Fcγ (applied apically at pH 5.9) and AlexaFluor-488-labeled pIgA (applied basolaterally at pH 7.4) were incubated with FcRn-pIgR-MDCK cells for a short pulse and then chased for the indicated times. Fixed cells were examined by confocal microscopy as whole cell volumes [panels A and C (top)] or sub-volumes (panels B–E). Bars (A and B) = 2.5 µm. Quantitative 3-D colocalization analyses are presented as Pearson's correlation coefficients. Values in histograms represent the mean and standard error from measurements taken from 11 to 19 cells per condition. Calculated p-values for the statistical significance of differences between pairwise combinations in the histograms are presented in Table S1. (A) Left: Side-views of FcRn-pIgA-MDCK cells at indicated chase times showing Fcγ (red) and pIgA (green) fluorescence. Regions of colocalization appear yellow. Right: Schematic diagram to define three sub-volumes: apical (Ap), medial (MED) and basolateral (BL). (B) Confocal data from panel A displayed as sub-volumes projected down the apical-to-basolateral direction (*Z*-axis in panel A). (C) Quantitative analyses to compare Fcγ/pIgA colocalization as a function of chase time in whole cell volumes (top) or sub-volumes (bottom). (D) Percent of total Fcγ fluorescence in sub-volumes as a function of chase time. (E) Percent of total pIgA fluorescence in sub-volumes as a function of chase time.

To compare apical-to-basolateral transport by FcRn and basolateral-to-apical transport by pIgR, the apical surface of FcRn-pIgR-MDCK cells was incubated with fluorescently labeled Fcγ at pH 5.9 while the basolateral surface was incubated with labeled pIgA at pH 7.4. After the labeled ligands were chased for 2–30 min, the cells were fixed and examined by confocal microscopy (Figure 1). Three-dimensional (3-D) confocal image stacks were analyzed quantitatively for the amounts of Fcγ and pIgA fluorescence and for the degree of overlap between Fcγ and pIgA as a function of chase time. Side-views of Fcγ and pIgA fluorescence in a reconstructed whole cell volume from FcRn-pIgR-MDCK cells (Figure 1A) show that fluorescence from each ligand was initially localized primarily to regions near the surface where it was applied; hence, there was little opportunity for colocalization at early time-points. However, as both ligands redistributed to other regions of the cell at later time-points (Figure 1B), they showed stronger colocalization that peaked at 20 min of chase (Figure 1C, top), suggesting that the trafficking routes of Fcγ and pIgA intermix over time. The p-values for the statistical significance of differences between all pairwise combinations in this and all other histograms are presented in Table S1.

To better analyze transport and colocalization, whole cell volumes were parsed into three sub-volumes: Ap, the ‘top’ one-third of the cell as oriented in Figure 1A (includes the apical surface and cytoplasm above and just below the tight junctions); MED, the ‘medial’ one-third of the cell; and BL, the ‘bottom’ one-third of the cell. As all of the plasma membranes ‘below’ the tight junctions are basolateral membranes, both the MED and BL sub-volumes include basolateral membrane that could serve as an exit point for apically applied Fcγ or as an entry point for basolaterally applied pIgA. Quantification of fluorescence as a function of chase time showed that Fcγ fluorescence started out mainly in the Ap sub-volume, but then spread to a roughly equivalent distribution across all three sub-volumes, never concentrating in the BL sub-volume (Figure 1D). This result is consistent with the observations from our electron tomography study of gold-labeled Fcγ transport by FcRn in the rat neonatal small intestine (49), which showed that the majority of labeled ligands were found in the ‘top’ half (as defined by Figure 1A) of polarized epithelial cells, with less labeled ligands in the ‘bottom’ half of the cells. In contrast to FcRn-mediated transport of Fcγ, the confocal image stacks revealed that pIgA transport is strongly unidirectional: the majority of pIgA was concentrated in the BL sub-volume at short chase times, but shifted to a concentration in the Ap sub-volume at the 20- and 30-min chase times (Figure 1E) (p < 0.05 for differences in pIgA fluorescence at the 2- and 30-min time-points in each of the three sub-volumes; Table S1).

**Figure 2:**
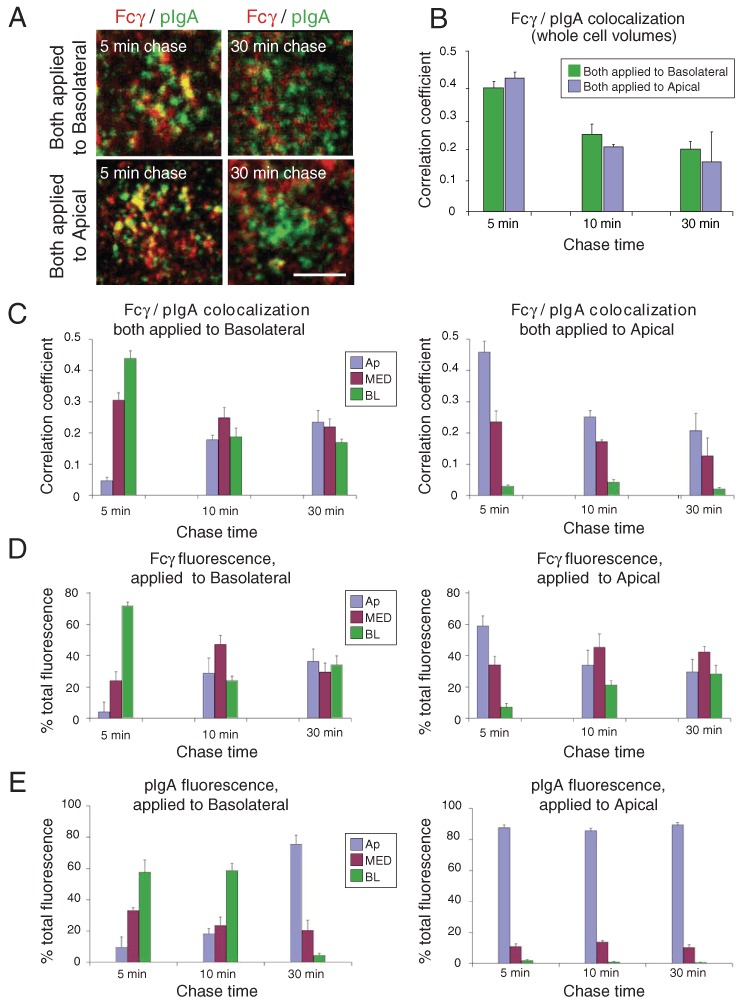
**Colocalization of Fcγ and pIgA when both applied to the same surface.** AlexaFluor-568-labeled Fcγ and AlexaFluor-488-labeled pIgA were both incubated with either the apical surface or the basolateral surface of FcRn-pIgR-MDCK cells for a short pulse at pH 5.9 and then chased for the indicated times. Quantitative 3-D colocalization analyses are presented as Pearson's correlation coefficients. Values in histograms represent the mean and standard error from measurements taken from 11 to 19 cells per condition. Calculated p-values for the statistical significance of differences between pairwise combinations in the histograms are presented in Table S1. (A) Whole cell volume projections are shown with Fcγ fluorescence in red, pIgA fluorescence in green, and regions of colocalization in yellow. Bar = 2.5 µm. (B) Quantitative 3-D colocalization analysis to compare Fcγ/pIgA colocalization as a function of chase time in whole cell volumes when both ligands were applied to the basolateral or apical surface. (C) Quantitative 3-D colocalization analysis to compare Fcγ/pIgA colocalization as a function of chase time in sub-volumes when both ligands were applied to the basolateral (left) or apical (right) surface. (D) Percent of total Fcγ fluorescence in sub-volumes as a function of chase time when Fcγ was applied to the basolateral (left) or apical (right) surface. (E) Percent of total pIgA fluorescence in sub-volumes as a function of chase time when pIgA was applied to the basolateral (left) or apical (right) surface.

Because FcRn engages in bidirectional transcytosis (29–31,40), we could study FcRn-mediated transport in FcRn-expressing cells after labeled Fcγ protein was incubated at either the apical or basolateral surface. Furthermore, we could investigate trafficking of Fcγ and pIgA when both ligands were applied to the basolateral surface of FcRn-pIgR-MDCK cells. Although pIgR mediates only basolateral-to-apical transcytosis, uncleaved pIgR can endocytose apically applied pIgA (16); thus, we could also investigate the degree to which Fcγ and pIgA colocalized three-dimensionally when both ligands were applied to the apical surface. Strong colocalization of labeled Fcγ and pIgA was observed at the 5-min chase time-point after they were co-incubated at acidic pH at either the basolateral or the apical surface of polarized FcRn-pIgR-MDCK cells (Figure 2A–C), suggesting endocytosis of both ligands into the same population of endosomes. At later time-points, the colocalization dropped significantly (Table S1), indicating that Fcγ and pIgA pathways diverged even when endocytosis occurred on the same surface. Evaluation of the fluorescence signal for each ligand within each of the three sub-volumes (Figure 2D,E) showed that Fcγ and pIgA achieved distributions characteristic of their cognate receptors over time: the bidirectional receptor FcRn shifted the distribution of Fcγ evenly throughout the cell, whereas the unidirectional receptor pIgR shifted the distribution of pIgA to the apical region, regardless of the surface from which the ligand was internalized. This is consistent with the idea that Fcγ can be transcytosed to the opposite surface when applied either apically or basolaterally, whereas pIgA will be transcytosed only when applied to the basolateral surface because uncleaved pIgR that endocytoses pIgA from the apical surface would primarily return it to that surface rather than transporting it to the basolateral surface (16). Whether the Fcγ that colocalized with pIgA in apical regions shortly after apical loading was committed to being recycled (as pIgA would be) was not entirely clear, as recent evidence suggests that recycling endosomes can serve as staging grounds for the entry of FcRn into transcytotic or recycling pathways (37–42).

### Three-dimensional colocalization of Fcγ and pIgA in apical early endosomes during transcytosis

In the previous studies with FcRn-MDCK cells, we showed that a portion of Fcγ internalized under steady-state conditions was present in compartments that labeled with early endosome antigen-1 (EEA1), a marker for early endosomes (40). Here we used FcRn-pIgR-MDCK cells to investigate the kinetics of Fcγ and pIgA passage through EEA1-positive endosomes, analyzing both whole cell volumes and Ap, MED, and BL sub-volumes. FcRn-pIgR-MDCK cells were incubated with fluorescently labeled Fcγ and/or pIgA, chased for 2–30 min, and then fixed and stained with an anti-EEA1 antibody.

**Figure 3:**
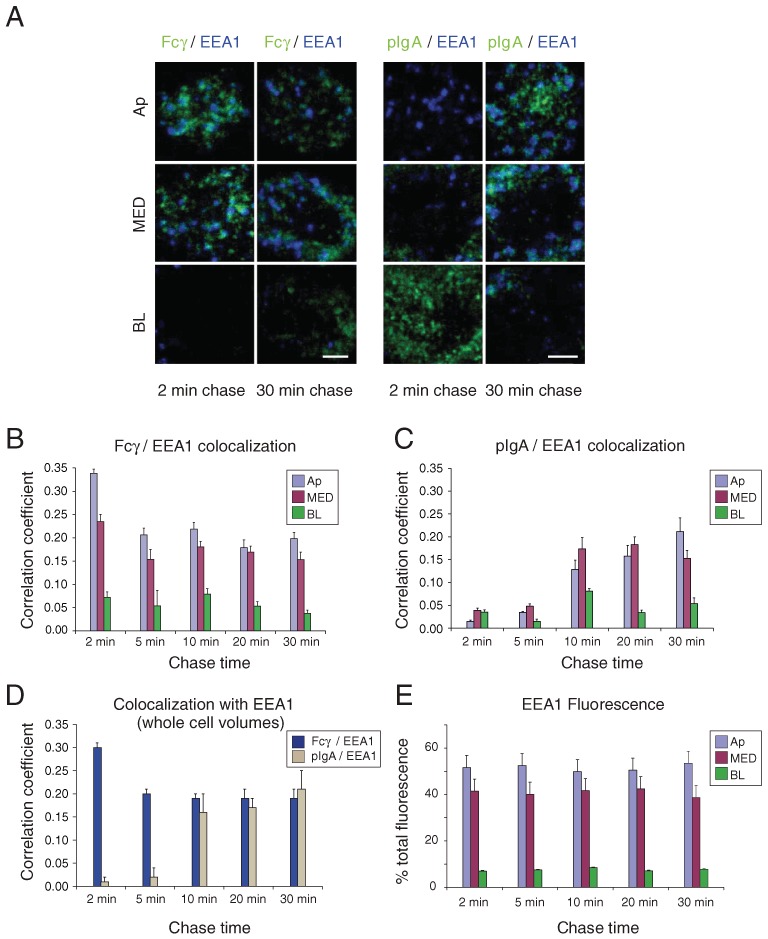
**Colocalization of Fcγ and pIgA with EEA1.** AlexaFluor-488-labeled Fcγ (applied apically at pH 5.9) or AlexaFluor-488-labeled pIgA (applied basolaterally at pH 7.4) was incubated with FcRn-MDCK or pIgR-MDCK cells for a short pulse and then chased for the indicated times. Fixed cells were prepared for immunofluorescence using an antibody against EEA1 and examined by confocal microscopy. Quantitative 3-D colocalization analyses are presented as Pearson's correlation coefficients. Values in histograms represent the mean and standard error from measurements taken from 11 to 19 cells per condition. Calculated p-values for the statistical significance of differences between pairwise combinations in the histograms are presented in Table S1. (A) Fcγ or pIgA fluorescence (green) is shown with EEA1 fluorescence (blue) in the indicated sub-volumes. Regions of colocalization appear in cyan. Bar = 2.5 µm. (B) Quantitative 3-D analysis of Fcγ/EEA1 colocalization as a function of chase time in sub-volumes. (C) Quantitative 3-D analysis of pIgA/EEA1 colocalization as a function of chase time in sub-volumes. (D) Quantitative 3-D analysis of Fcγ/EEA1 and pIgA/EEA1 colocalization as a function of chase time in whole cell volumes. (E) Percent of total EEA1 fluorescence in sub-volumes as a function of chase time.

Figure 3A compares 3-D colocalization of EEA1 with Fcγ and pIgA after various chase times. At the earliest chase time (2 min), Fcγ was strongly colocalized with EEA1 in the Ap sub-volume, but colocalization decreased in the Ap sub-volume at later chase times (Figure 3B) (p < 0.05 for 2–5 min and 2–30 min pairwise comparisons; Table S1). The degree of colocalization remained at a fairly constant level for chase times after 2 min, most likely because of apical surface recycling events involving EEA1-positive endosomes (Figure 3B,D). There was less colocalization of Fcγ with EEA1 in the BL sub-volume, and somewhat less in the MED sub-volume, at all time-points (Figure 3B; p < 0.05; Table S1). In contrast, colocalization of pIgA with EEA1-positive endosomes was minimal in all parts of the cell at the earliest chase times, but rose to higher levels by the longest chase times (Figure 3C,D). There was minimal colocalization of EEA1 with pIgA in the BL sub-volume at any time-point. The significantly higher colocalization at later time-points in the Ap and MED sub-volumes (p < 0.05; Table S1) most likely represents transport of pIgA into apical early endosomes prior to exocytosis at the apical plasma membrane. Comparison of Fcγ versus pIgA colocalization with EEA1 in whole cell volumes (Figure 3D) clearly summarizes differences in the trafficking of the two ligands: colocalization with Fcγ is the highest at the earliest chase time, then falls to a lower but constant level, whereas colocalization with pIgA is insignificant at the earliest chase time, then rises steadily to its highest level at the 30-min chase time (Table S1). The majority of EEA1 fluorescence was found in the Ap sub-volume, with some fluorescence within the MED and Bl sub-volumes, consistent with EEA1 being a marker for both apical and basolateral early endosomes (13). As expected, the distribution of EEA1 within the three sub-volumes did not change as a function of chase time (Figure 3E; Table S1).

When both ligands were added simultaneously to FcRn-pIgR-MDCK cells (Fcγ to the apical surface, pIgA to the basolateral surface), a population of triple-positive endosomes (EEA1-, Fcγ- and pIgA-positive) became enriched over time (Figure S1), demonstrating intermixing of the FcRn-Fcγ and pIgR–pIgA trafficking pathways in early endosomal compartments. Quantitative colocalization analyses of whole cell volumes (Figure S1A) showed that Fcγ entered EEA1-positive endosomes at early time-points and decreased in colocalization only slightly by the 20-min time-point, whereas there was minimal colocalization of pIgA with EEA1 immediately after basolateral internalization, but pIgA entered EEA1-positive endosomes at later time-points. An increase in colocalization of Fcγ with EEA1 at early chase times (2 min, 5 min) (Figures 3A,B,D and S1; Table S1) suggests that FcRn–Fcγ complexes traveled through early and/or sorting endosomes immediately following internalization, whereas the gradually increasing colocalization of pIgA with the same markers (Figures 3A,C,D and S1; Table S1) suggests that a significant portion of basolaterally internalized pIgR did not enter these structures until it arrived at the apical domain of the cell, where EEA1-positive endosomes were more abundant (Figure 3E).

### Three-dimensional colocalization of Fcγ and pIgA in recycling endosomes during transcytosis

**Figure 4:**
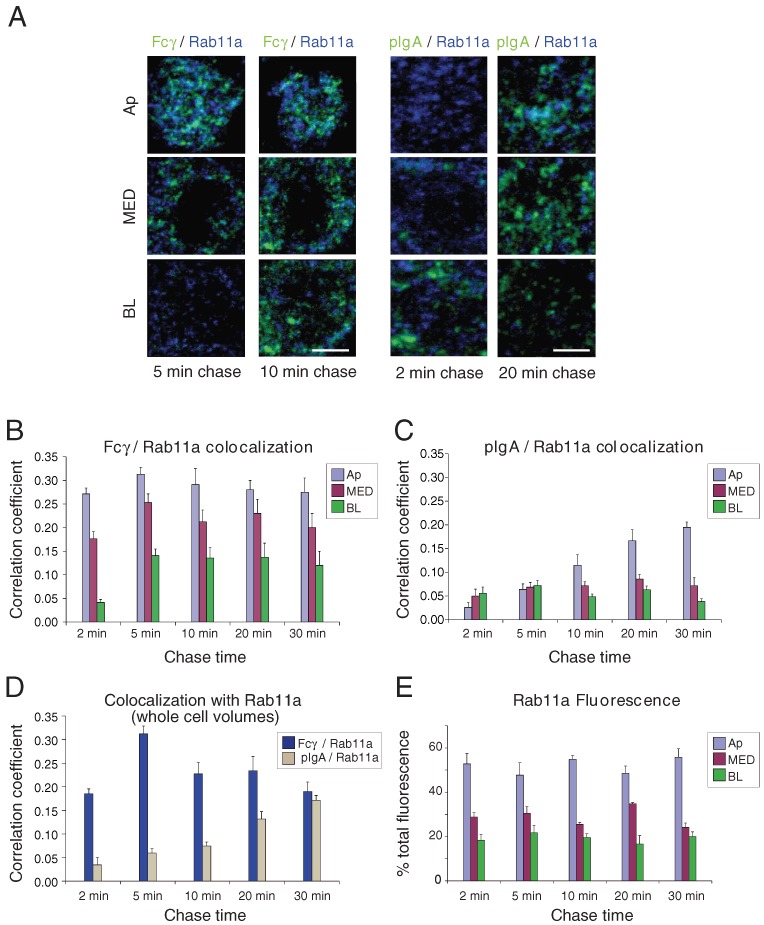
**Colocalization of Fcγ and pIgA with Rab11a.** AlexaFluor-488-labeled Fcγ (applied apically at pH 5.9) or AlexaFluor-488-labeled pIgA (applied basolaterally at pH 7.4) was incubated with FcRn-MDCK or pIgR-MDCK cells for a short pulse and then chased for the indicated times. Fixed cells were prepared for immunofluorescence using an antibody against Rab11a and examined by confocal microscopy. Quantitative 3-D colocalization analyses are presented as Pearson's correlation coefficients. Values in histograms represent the mean and standard error from measurements taken from 11 to 19 cells per condition. Calculated p-values for the statistical significance of differences between pairwise combinations in the histograms are presented in Table S1. (A) Fcγ or pIgA fluorescence (green) is shown with Rab11a fluorescence (blue) in the indicated sub-volumes. Regions of colocalization appear in cyan. Bar = 2.5 µm. (B) Quantitative 3-D analysis of Fcγ/Rab11a colocalization as a function of chase time in sub-volumes. (C) Quantitative 3-D analysis of pIgA/Rab11a colocalization as a function of chase time in sub-volumes. (D) Quantitative 3-D analysis of Fcγ/Rab11a and pIgA/Rab11a colocalization as a function of chase time in whole cell volumes. (E) Percent of total Rab11a fluorescence in sub-volumes as a function of chase time.

Rab11a is a commonly used marker for recycling endosomes (50,51), and recycling endosomes that are positive for both Rab11a and Rab25 are known to be involved in both FcRn- and pIgR-mediated trafficking (42,52). For comparison with earlier FcRn-based studies that used Rab11a as a recycling endosome marker (46,49), we conducted experiments analogous to the EEA1 colocalization studies described above using an anti-Rab11a antibody. At early time-points Fcγ colocalized with Rab11a, with the highest colocalization found in the Ap and MED sub-volumes (Figure 4A,B; Table S1). Colocalization remained fairly constant throughout the chase times in these sub-volumes, whereas there was little or no colocalization in the BL sub-volume until the 5-min and later time-points (Figure 4B). By contrast, pIgA and Rab11a showed only low levels of colocalization at early time-points in all three sub-volumes, and it was not until pIgA had traversed the cell to the Ap sub-volume that higher levels of colocalization were seen (after ≥10 min of chase time) (Figure 4C; Table S1). The comparison of Fcγ versus pIgA colocalization with Rab11a as a function of chase time in whole cell volumes (Figure 4D) once again summarizes differences in FcRn- and pIgR-mediated transport: Fcγ colocalization remained fairly constant throughout the chase times, with a peak at the 5-min chase time for the whole cell volumes, whereas pIgA colocalization rose steadily and peaked only at the latest chase time (Table S1). Quantification of Rab11a staining throughout the three sub-volumes indicated that about half of Rab11a-positive vesicles were located in the Ap sub-volume, while the other half resided in the MED and BL sub-volumes (Figure 4E). Similar to our conclusions for the EEA1 colocalization experiments (Figures 3 and S1), these results suggest that FcRn transported its ligands to Rab11a-positive sorting/recycling endosomes quickly following apical internalization (Figure 4A,B,D), whereas pIgR did not traffic its basolaterally internalized ligands to these structures until it had entered the apical domain (Figure 4A,C,D), where these structures were more abundant (Figure 4E).

Recent functional studies demonstrated that gene suppression of Rab25, but not Rab11a, affected FcRn-mediated transcytosis; thus, Rab25 was required for a sorting step specific for transcytosis, but not recycling, whereas Rab11a regulated recycling to the basolateral membrane but was not required for transcytosis (42). These results suggest that the Fcγ+/Rab11+ vesicles we observed represent recycling endosomes (primarily apical recycling endosomes) involved in Fcγ+ recycling rather than transcytosis.

### Three-dimensional colocalization of Fcγ and pIgA with Tf and late endosomal/lysosomal markers

**Figure 5:**
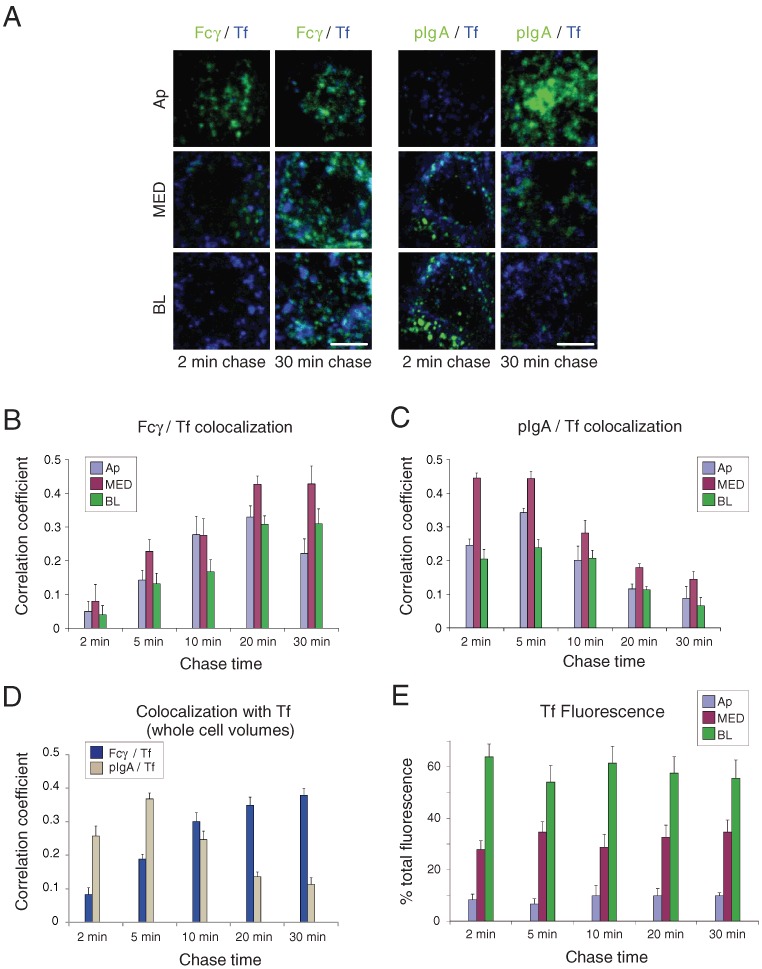
**Colocalization of Fcγ and pIgA with Tf.** AlexaFluor-488-labeled Fcγ (applied apically at pH 5.9) or AlexaFluor-488-labeled pIgA (applied basolaterally at pH 7.4 together with AlexaFluor-568- or -647-labeled Tf) was incubated with FcRn-MDCK or pIgR-MDCK cells for a short pulse and then chased for the indicated times. Cells were fixed and examined by confocal microscopy. Quantitative 3-D colocalization analyses are presented as Pearson's correlation coefficients. Values in histograms represent the mean and standard error from measurements taken from 11 to 19 cells per condition. Calculated p-values for the statistical significance of differences between pairwise combinations in the histograms are presented in Table S1. (A) Fcγ or pIgA fluorescence (green) is shown with Tf fluorescence (blue) in the indicated sub-volumes. Regions of colocalization appear in cyan. Bar = 2.5 µm. (B) Quantitative 3-D analysis of Fcγ/Tf colocalization as a function of chase time in sub-volumes. (C) Quantitative 3-D analysis of pIgA/Tf colocalization as a function of chase time in sub-volumes. (D) Quantitative 3-D analysis of Fcγ/Tf and pIgA/Tf colocalization as a function of chase time in whole cell volumes. (E) Percent of total Tf fluorescence in sub-volumes as a function of chase time.

The colocalization studies with EEA1 and Rab11a described above allowed us to observe trafficking of Fcγ and pIgA in the context of apical early and recycling endosomes. Iron-loaded Tf internalized by the Tf receptor was used to compare Fcγ and pIgA trafficking in basolateral portions of the cell. Tf is a marker for basolateral recycling endosomes, common recycling endosomes, and apical endosomes that lack Rab11a (13,53). Fluorescently labeled Tf was incubated at pH 7.4 at the basolateral surface and labeled Fcγ was incubated at pH 5.9 at the apical surface of FcRn-MDCK cells, or labeled pIgA was incubated together with Tf at the basolateral surface of pIgR-MDCK cells (Figure 5). Fcγ colocalization increased as a function of chase time, whereas pIgA colocalization decreased after the 5-min chase time (Figure 5A–D; Table S1). This suggests that Fcγ reached Tf-positive compartments at later trafficking steps (Figure 5B,D), whereas basolaterally internalized pIgA initially entered, and was later sorted away from Tf-positive compartments (Figure 5C,D). This is consistent with the observation that Tf-positive structures were enriched in the basolateral region of the cell (Figure 5E) and that apically internalized Fcγ would require more time to be transported to this region. The Fcγ-Tf and pIgA-Tf colocalization patterns followed opposite temporal trends than colocalizations of Fcγ and pIgA with the apical markers EEA1 and Rab11a: Fcγ colocalization with the apical markers was higher at earlier chase times, but was higher at later times with Tf. Conversely, pIgA colocalization was higher at later chase times with the apical markers but at earlier times with Tf.

Similar colocalization experiments were conducted using antibodies against late endosomal markers (anti-Rab7 and anti-Rab9) and an antibody against LAMP2, a lysosomal marker. As previously observed (40), we detected only minimal colocalization of Fcγ with late endosomal or lysosomal markers (Figure S2A), consistent with FcRn's function in rescuing Fcγ and IgG from degradation in late endosomes and lysosomes (22–24). Similarly, pIgA showed minimal colocalization with late endosomal or lysosomal markers (Figure S2B).

### Fcγ and pIgA transport were equivalently affected by microtubule disruption

Disruption of the microtubule network in MDCK and other polarized cells has been shown to affect transport by pIgR, FcRn and other receptors to varying extents (16,43,54–56). However, our electron tomography investigation of gold-labeled Fcγ transport in neonatal intestinal cells revealed that it was often the case that only one of a group of individual Fcγ-containing vesicles within an entangled network was close enough to a microtubule to be linked via a motor protein, and that some Fcγ-containing vesicles were apparently not associated with a microtubule (49). These results suggested that disruption of microtubules decreases some, but not all, of the movement of Fcγ-containing vesicles. In order to examine the extent to which Fcγ transport depended upon intact microtubules and compare results with pIgA transport, we used nocodazole to disrupt cytoplasmic microtubules and observed the effects on the dynamics of Fcγ- and pIgA-positive vesicles.

Cells were pretreated with nocodazole for 1 h, and then incubated with labeled Fcγ (applied to the apical surface) or pIgA (applied to the basolateral surface) for 10 min in nocodazole-containing media. Control cells were subjected to the same treatments in the absence of nocodazole. Staining with an anti-tubulin antibody verified disruption of microtubules under the nocodazole conditions (data not shown). The trajectories of Fcγ- or pIgA-positive vesicles were then tracked in live cells. We interpret the fluorescent puncta that were tracked in the live-cell imaging experiments, which varied in apparent size from 200 to 600 nm, as groups of vesicles moving together as a unit. This interpretation is based on electron tomography results suggesting that vesicles containing FcRn–Fcγ complexes traveled as intertwined and tangled clumps along microtubule tracks and that individual Fcγ-containing vesicles had diameters as small as 60 nm (49), which would not be individually resolved in our experiments using conventional fluorescence microscopy (57).

**Figure 6:**
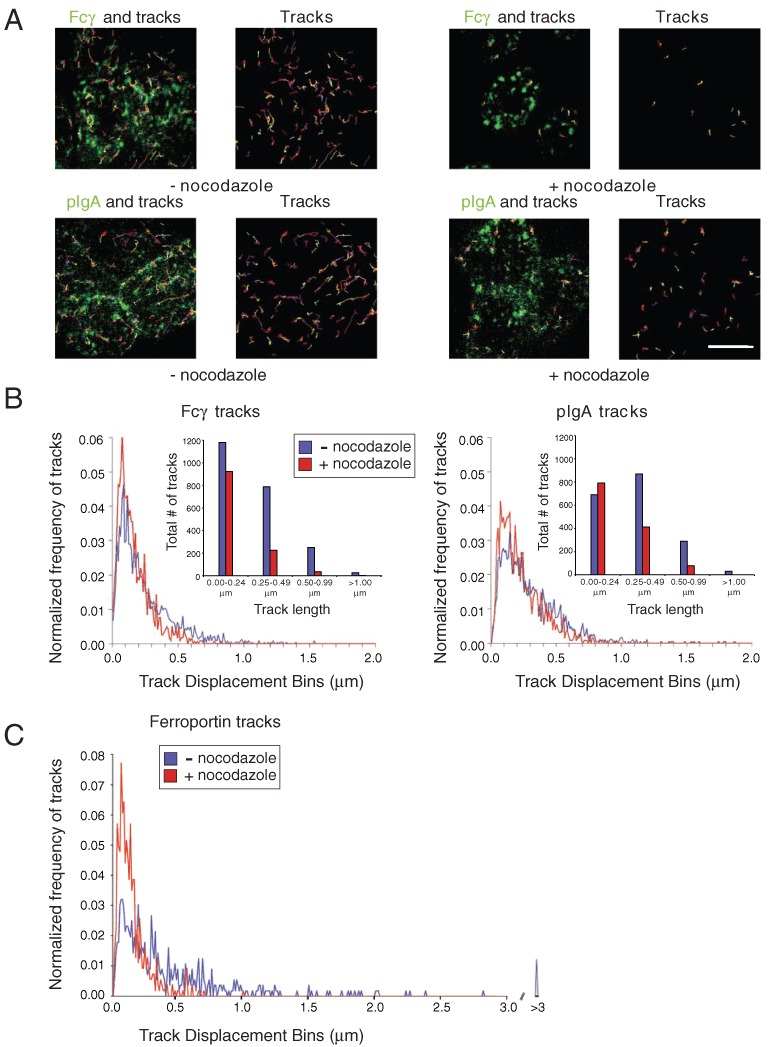
**Effects of nocodazole on the dynamics of Fcγ and pIgA-containing vesicles.** FcRn-MDCK or pIgR-MDCK cells were pretreated for 1 h with nocodazole-containing media or control media at 4°C. AlexaFluor-488-labeled Fcγ (applied apically at pH 5.9) or AlexaFluor-488-labeled pIgA (applied basolaterally at pH 7.4) was added to each condition and the incubation was continued for 10 min. Cells were imaged live after washing in the presence or absence of nocodazole. (A) Tracks (≥0.5 µm only) throughout a 30-second time-course were overlaid on the representative images or shown alone. Bar = 5 µm. (B) Normalized track displacements for Fcγ- and pIgA-positive tracked vesicles in untreated (blue) and nocodazole-treated (red) cells presented as histograms. Insets: binned data showing the total number of tracks in each of the four length categories. Note that there were 1.5- to 1.9-fold more trackable vesicles in the untreated samples compared to the nocodazole-treated samples (see *Methods*). (C) Normalized track displacements for ferroportin-positive tracked vesicles in untreated (blue) and nocodazole-treated (red) cells (similar to Figure 8b in ref 58, but presenting data for a 30-second time-course to correspond to time-courses in panel B).

Tracking of Fcγ and pIgA-positive vesicles in live cells demonstrated a decrease both in the total numbers of vesicles with trackable trajectories as well as in their track lengths in the nocodazole-treated cells compared to the control cells (Figure 6A). Histograms of tracked vesicles, normalized as a function of track length (Figure 6B), and presented as binned data for total numbers of tracked vesicles in each category of track length (insets to Figure 6B), demonstrated a comparable decrease in mobility for Fcγ- and pIgA-positive vesicles because of microtubule disruption. Although nocodazole affected the mobilities of both Fcγ- and pIgA-positive vesicles, the effects were not as large as we observed in a similar nocodazole-based study involving endocytosis and subsequent lysosomal targeting of a different receptor. In those experiments, we examined the mobilities of intracellular vesicles containing the iron export protein ferroportin, which had been internalized from the plasma membrane into endosomes and lysosomes after binding to the hormone hepcidin (58) (Figure 6C). The effects of disrupting microtubules were more substantial on ferroportin-positive vesicles than on Fcγ- and pIgA-positive vesicles, implying ferroportin-positive vesicles were more dependent upon microtubules for mobility. This interpretation is consistent with a notable population of ferroportin-positive vesicles with very long track displacements in untreated cells (Figure 6C). The differences in mobilities and microtubule dependencies between ferroportin-positive vesicles and Fcγ- or pIgA-positive vesicles may relate to differences in their trafficking itineraries and destinations: ferroportin does not undergo transcytosis; rather it is destined for degradation in lysosomes upon hepcidin-induced internalization (59), whereas Fcγ and pIgA are routed away from lysosomes and other degradative compartments by their transcytotic receptors (Figure S2).

**Figure 7:**
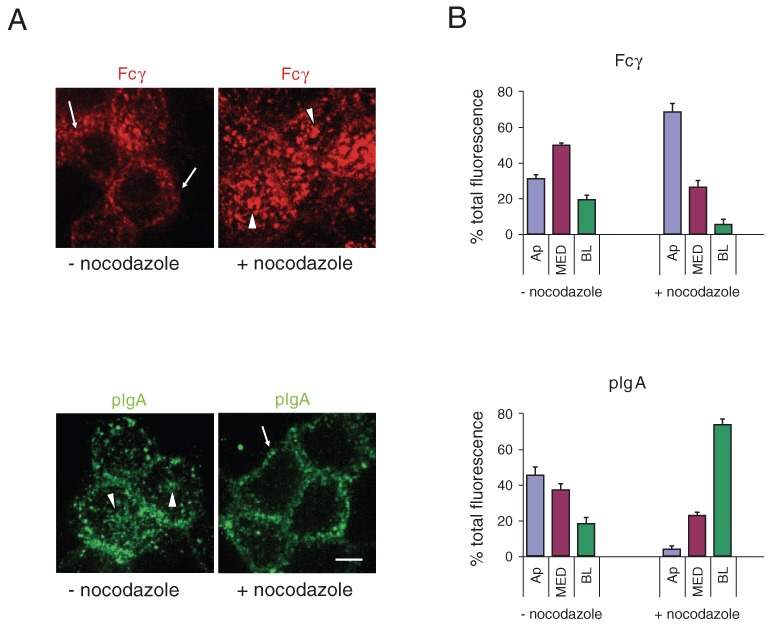
**Effects of nocodazole on the distribution of Fcγ and pIgA.** FcRn-MDCK or pIgR-MDCK cells were pretreated for 1 h with nocodazole-containing media or control media at 4°C. AlexaFluor-568-labeled Fcγ (applied apically at pH 5.9) or AlexaFluor-488-labeled pIgA (applied basolaterally at pH 7.4) was added to each condition and the incubation was continued for 20 min. Cells were fixed and examined by confocal microscopy. (A) Whole cell volume projection images. Arrowheads indicate apical staining; arrows indicate basolateral staining. Bar = 5 µm. (B) Percent of total Fcγ and pIgA fluorescence in sub-volumes of control and nocodazole-treated cells.

We next examined Fcγ and pIgA fluorescence in whole cell volumes and sub-volumes of fixed, nocodazole-treated and control cells in order to ascertain the effects of microtubule disruption on overall trafficking routes. As done in the live-cell imaging experiments, apically applied Fcγ or basolaterally applied pIgA was incubated with nocodazole-treated or control cells. Figure 7A shows overlay projections for whole cell volumes after a 20-min incubation with the labeled ligand in the presence or absence of nocadazole. Consistent with the reduced vesicle mobility observed by live imaging (Figure 6A,B), the distribution of labeled ligands showed marked differences in nocodazole-treated versus control cells. Fcγ-incubated control cells contained labeled vesicles throughout the cell volume, including the basolateral region, whereas the majority of vesicles in the nocodazole-treated cells were concentrated in the apical region (Figure 7B; Table S). The reverse was found for pIgA: pIgA-incubated control cells contained labeled vesicles throughout the cell volume, including the apical region, but the majority of vesicles in nocodazole-treated cells were concentrated basolaterally, with very few pIgA-positive vesicles in the Ap sub-volume (Figure 7B; Table S1). These results suggest that, although vesicles are capable of some movement, presumably by diffusion, when microtubules are disrupted, intact microtubules are required for most Fcγ- and pIgA vesicles to actively reach their target destinations.

## Conclusions

Our previous studies of FcRn trafficking (40,49) provided static information, which we have now expanded upon with live-cell imaging and pulse-chase analyses of fixed cells and simultaneous comparisons with another transcytosing receptor, pIgR, expressed in the same cells. This direct comparison of the two receptors permitted us to define how and where FcRn and pIgR utilized common sorting steps during the transport of their cognate ligands, IgG and pIgA, respectively. We found that Fcγ and pIgA met in some of the same vesicles as they crossed cell monolayers, whether applied at the same or at opposite surfaces (Figures 1 and 2). These results demonstrated similarities in their trafficking routes, but immunolabeling analyses using endosomal markers also revealed differences consistent with FcRn's functions in bidirectional transcytosis and recycling and pIgR's function in unidirectional transcytosis. (Figures 3–5). The finding that Fcγ- and pIgA-positive vesicles exhibited similar mobility characteristics and were equivalently affected by nocodazole (Figures 6 and 7) suggests that FcRn- and pIgR-dependent trafficking are likely regulated by some of the same trafficking machinery, which may have different properties from the machinery used for non-transcytotic itineraries—specifically, the transport of vesicles containing proteins targeted for lysosomal degradation (Figure 6C). The use of polarized cells expressing two well-characterized transcytotic receptors represents an advance that can be used for further studies to thoroughly characterize endocytosis, recycling and both apical-to-basolateral and basolateral-to-apical transcytosis.

## Materials and Methods

### Reagents and antibodies

Fc fragments show no significant differences from intact IgG in binding to rat FcRn or uptake/transcytosis by rat FcRn (40,60); hence, rat Fcγ (the Fc fragment from a rat IgG2a) was used as the FcRn ligand in these experiments. Rat Fcγ was expressed in Chinese hamster ovary (CHO) cells and purified as previously described (61). Human polymeric IgA, a ligand for pIgR, was kindly provided by Jean-Pierre Vaerman (Catholic University of Louvain, Brussels, Belgium) (62,63). Fcγ and pIgA were conjugated to AlexaFluor-488 or -568 using a Protein Labeling Kit (Molecular Probes, Inc.) and then separated from unconjugated dye according to the manufacturer's instructions. The concentration and degree of labeling were determined spectrophotometrically using published extinction coefficients at 280 nm. Canine apo-Tf (Sigma-Aldrich) was loaded with iron by incubation with bicarbonate and excess ferric ammonium sulfate and then directly conjugated to AlexaFluor-568 or -647 as described above.

Primary antibodies used for colocalization experiments were obtained as follows: mouse monoclonal anti-EEA1 from BD Transduction Laboratories; rabbit polyclonal anti-Rab11a from Invitrogen Inc.; mouse monoclonals anti-Rab7 and Rab9 from Sigma-Aldrich; mouse monoclonal anti-α-tubulin from Molecular Probes, Inc.; mouse monoclonal anti-secretory component from Abcam, Inc. A mouse monoclonal antibody (AC17) against canine LAMP-2 was a gift from Dr E. Rodriguez-Boulan (Weill Medical College, Cornell University). Anti-EEA1, -Rab11a, -Rab7, -Rab9 antibodies were directly conjugated to AlexaFluor-647 as described above. The remaining antibodies were indirectly labeled using a goat anti-mouse AlexaFluor-568 or -468 secondary antibody (Molecular Probes, Inc.).

### Cell culture

FcRn-MDCK, a previously described stable MDCK II cell line expressing rat FcRn and rat ß2-microglobulin (the FcRn light chain) (40), was cultured in minimum essential medium (MEM, GibcoBRL/Invitrogen) supplemented with 10% FBS (HyClone) and 0.25 mg/mL G418 (Invitrogen) at 37°C, 5% CO_2_. To generate polarized cell monolayers, cells were seeded at approximately 250,000 cells/filter onto 12-mm Transwell polyester filters (Corning Costar). The apical and basolateral reservoirs were filled with 0.5 and 1.5 mL of media, respectively. Cells were used for experiments on the fourth or fifth day after plating.

### Generation of FcRn-pIgR-MDCK and pIgR-MDCK cells

The gene encoding human pIgR was incorporated into the XbaI and AscI restriction sites of the lentiviral vector c-FUW (64). A c-FUW vector containing the gene encoding EGFP (enhanced green fluorescent protein) was used as a positive control to make EGFP-expressing viruses for infections. 293FT cells were grown and maintained in MEM supplemented with 10% FBS, 500 µg/mL G418 (to maintain selection of cells expressing T-antigen), and pen-strep. One day prior to transfection to produce recombinant lentiviruses, cells were trypsinized and seeded to a 10-cm plate in medium without G418 so as to be approximately 90% confluent the following day. On the day of transfection, cells were washed with PBS and incubated in serum-free OptiMEM I approximately 1 h prior to transfection. Complexes were formed by combining 60 µL Lipofectamine 2000 (diluted into 1.5 mL serum-free OptiMEM) with 1.5 mL of OptiMEM containing 9.6 µg of the lentiviral vector and 4.3 µg each of helper plasmids REV, RRE and VSVg (64). After incubating at room temperature for 25 min, the media on the cells were aspirated and replaced with 12 mL fresh serum-free OptiMEM. The complexes (3 mL) were added dropwise to plates, which were mixed by gentle rocking and returned to 37°C. After 4–6 h, the complexes were removed and replaced with 10 mL of complete medium (MEM, 10% FBS, pen-strep) without G418. Viral supernatants were harvested 72 h later and passed through a 0.45-µm syringe filter to remove cellular debris. In most cases, viruses were used for infection immediately, and remaining viral supernatants were aliquoted and frozen at −80°C for later use.

Polarized MDCK or FcRn-MDCK cells were infected with pIgR- and EGFP-viral supernatants at 1:10, 1:100 or 1:1000 dilutions in normal growth media to determine viral titer. In general, the highest dilution that resulted in more than 50% infection was used for experiments. After addition of virus, cells were incubated overnight in a dedicated 37°C incubator and the medium was replaced the following day. pIgR expression was confirmed by immunofluorescence microscopy using an antibody against secretory component (data not shown).

### Incubation with Fcγ, pIgA and Tf

For studies of FcRn-mediated transport of Fcγ, fluorescently labeled Fcγ (27.5 mg/mL; 500 nM) in Hank's Buffered Saline Solution (HBSS)/10 mm MES pH 5.9 was internalized from the apical surface (or from the basolateral surface for the experiments shown in Figure 2), while the opposite surface was maintained in HBSS/10 mM Hepes pH 7.4. For studies of pIgR-mediated transport of pIgA, fluorescently labeled pIgA (100 mg/mL; approximately 300 nM) was internalized at pH 7.4 from the basolateral surface (or from the apical surface at pH 5.9 for the experiments shown in Figure 2). In pulse-chase experiments, unbound ligands were removed by washing with HBSS pH 7.4 after 2 min of incubation and then chased in the same buffer for 2, 5, 10, 20 or 30 min. In some experiments, Tf-positive compartments were labeled by incubating the basolateral surface of cells with fluorescently labeled Tf (20 mg/mL; 280 nm) at pH 7.4 for 15 min. pIgA was added to the basolateral surface or Fcγ was added to the apical surface during the last 2 min, and then the cells were washed and chased. For all timed experiments, filter plates were incubated at 37°C in a prewarmed chamber for the indicated times, after which the plates were placed on ice and processed for immunofluorescence as described below.

### Preparation of fixed cell samples

For immunofluorescence staining, cells were washed briefly with ice-cold PBS+ (PBS supplemented with 1 mm CaCl_2_, 0.5 mM MgCl_2_ and 0.25 mM MgSO_4_) and fixed using modifications of a previously described protocol (40). Briefly, cells were incubated for 15 min at room temperature in 4% paraformaldehyde (PFA) in PBS+. After quenching excess aldehyde with freshly prepared 100 mm glycine in PBS+ for 10 min, the cells were washed twice with PBS+ and blocked for 30 min at room temperature in PBS containing 10% normal goat serum and 0.025% saponin. Cells were then incubated for 1 h at room temperature in blocking buffer containing one or more of the following directly labeled primary antibodies: mouse anti-EEA1 (3–4 µg/mL), rabbit anti-Rab11a (7–8 µg/mL), mouse anti-Rab7 (7–8 µg/mL), or mouse anti-Rab9 (7–8 µg/mL); or the following unlabeled primary antibodies: mouse anti-α-tubulin (1 µg/mL), mouse anti-secretory component (10 µg/mL) or mouse anti-LAMP-2 (AC17) (20 µg/mL). Tubulin, pIgR and LAMP-2 immunostaining were done using unlabeled mouse antibodies and a goat anti-mouse AlexaFluor-568 or -468 diluted 1:300 in blocking buffer for 1 h at room temperature.

The cells were washed twice more with PBS and the filters were then removed from their holders using a scalpel and a fine-tipped pair of forceps. Excised filters were mounted on glass slides using ProLong Gold antifade medium (Invitrogen), and cured at room temperature overnight before confocal imaging.

For experiments involving nocodazole treatment, FcRn-MDCK or pIgR-MDCK cells were pretreated for 1 h with media containing 33 µM nocodazole (Sigma) or control media with no nocodazole at 4°C. Ligands (500 nM Fcγ applied to the apical surface at pH 5.9 or 300 nM pIgA applied to the basolateral surface at pH 7.4) were added to each condition and the incubation was continued for 20 min at 37°C. Cells were then fixed as described above.

### Confocal imaging and image processing

Confocal images were acquired using a synchronized UltraVIEW ERS Rapid Confocal Imager (Perkin-Elmer) connected to a Zeiss Axio Observer Microscope fitted with a 100× objective lens (αPlan-APOCHROMAT 1.46 Oil DIC, Zeiss). Alexa-488, -568 and -647 fluorophores were excited at 488, 568 or 647 nm, respectively, using a 488/548/647 multiline argon/krypton laser (Melles Griot). All fixed cells that were imaged had intact nuclei, as determined by 4′,6-diamidino-2-phenylindole (DAPI) staining (data not shown).

Three-dimensional volumes of z stacks (0.2 µm spacing between single confocal slices) were reconstructed using Imaris 6.0.1 (Bitplane, Inc.). Confocal micrographs shown are representative slices from 3-D confocal stacks, intact cell projections, or projections of sub-volumes composed of the ‘top’ (as oriented in Figure 1A) 33% of the cell volume (defined as apical, Ap, in the text and figures), the ‘middle’ 33% of the cell volume (defined as medial, MED) and the ‘bottom’ 33% of the cell volume (defined as basolateral, BL). The degree of colocalization was assessed three-dimensionally in whole cell volumes and sub-volumes by calculating the Pearson's correlation coefficient in the region of interest using a semi-automated algorithm embedded in the Coloc module of the Imaris 6.0.1 software (65). The Coloc module also performed a two-step analysis to calculate the Pearson's correlation coefficient for the original data and for a large set (approximately 200) of images randomized with a grain size determined by the point spread function of the microscope objective (65). If the Pearson's correlation coefficient of the original image was not greater than 95% of the randomized images, then the colocalization analysis did not continue. In addition, we did not use very bright or very weak images for the analyses, saturated or near-threshold signals were omitted, and user bias in setting analysis parameters was avoided by using an automated thresholding procedure (65).

Histograms presenting the mean correlation coefficient (derived from 11 to 19 cells assessed per treatment condition) are shown with standard error bars in all figures. Tests of statistical significance for differences between pairwise combinations were calculated using the two-tailed Student's *t*-test (Table S1). Representative confocal images from whole cell or sub-volume projections demonstrating colocalization or a lack of colocalization are shown in Figures 1–5, S1 and S2.

### Live-cell imaging

Polarized monolayers of FcRn-MDCK or pIgR-MDCK cells were grown on the bottom of permeable filter supports for 4–5 days (14). Cells on filter supports were pretreated with media containing 33 µM nocodazole or with control media lacking nocodazole for 1 h at 4°C. Subsequent ligand incubations and live-cell imaging experiments were performed at 37°C in the continued presence of the drug. For Fcγ uptake, FcRn-MDCK cells were incubated with labeled Fcγ (see above) at pH 5.9 for 10 min on the apical surface while the basolateral surface was maintained at pH 7.4. For pIgA uptake, pIgR-MDCK cells were incubated with labeled pIgA for 10 min at the basolateral surface and both surfaces maintained at pH 7.4. Following incubation with labeled ligands, cells were washed with PBS+, filters were transferred into glass-bottom dishes (MatTek) and were imaged in Leibovitz's L-15 Medium (Invitrogen) containing l-glutamine and no phenol red. Imaging was performed using an UltraVIEW ERS Rapid Confocal Imager equipped with a temperature-controlled housing (Solent Scientific).

To observe the dynamics of vesicle movement, single focal plane time lapses were collected for the period of 30 seconds or longer with 200-millisecond exposure times and with no delay between frames (resulting in a final acquisition speed of approximately 5 frames per second). Seven FcRn-positive cells and three pIgR-positive cells from each condition were imaged. Fcγ- or pIgA-containing compartments were tracked using the tracking module in Imaris 6.0.1 (Bitplane). Spots and tracks were automatically selected using the following Imaris parameters: estimated diameter = 0.3 µm; threshold = 6; Brownian motion tracking algorithm; max distance = 2.0 µm; and gap size = 3; with minimal manual removal required. Tracks shorter than three frames (0.63 seconds) were filtered out of the data set. The final data set included 2243 tracks for the untreated Fcγ sample, 1183 tracks for the nocodazole-treated Fcγ sample, 1882 tracks for the untreated pIgA sample and 1221 tracks for nocodazole-treated pIgA sample.

## Supporting Information

**Figure S1: Triple and pairwise colocalizations of Fcγ, pIgA and EEA1 in FcRn-pIgR-MDCK cells.** AlexaFluor-568-labeled Fcγ (applied apically at pH 5.9) and AlexaFluor-488-labeled pIgA (applied basolaterally at pH 7.4) were incubated with FcRn-pIgR-MDCK cells for a short pulse and then chased for the indicated times. Fixed cells were prepared for immunofluorescence using an antibody against EEA1 and examined by confocal microscopy. Quantitative 3-D colocalization analyses are presented as Pearson's correlation coefficients. Values in histograms represent the mean and standard error from measurements taken from 11 to 19 cells per condition. Calculated p-values for the statistical significance of differences between pairwise combinations in the histograms are presented in Table S1. Bars (A and B) = 2.5 µm. (A) Left: Fcγ (red), pIgA (green) and EEA1 (blue) fluorescence are shown in whole cell volume projections. Regions of Fcγ/pIgA colocalization appear in yellow; Fcγ/EEA1 colocalization in magenta, pIgA/EEA1 colocalization in cyan and triple colocalization in white. Right:Quantitative colocalization analysis of Fcγ/EEA1, pIgA/EEA1and Fcγ/pIgA/EEA1 colocalization as a function of chase time in whole cell volumes. (B) Fcγ, pIgA and EEA1 fluorescence (colors as in panel A) are shown in sub-volumes for 5- and 20-min chase times. (C) Quantitative 3-D colocalization analyses of Fcγ/EEA1, pIgA/EEA1 and Fcγ/pIgA/EEA1 colocalization for 5- and 20-min chase times.

**Figure S2: Colocalization of Fcγ and pIgA with late endosomal and lysosomal markers.** AlexaFluor-488-labeled Fcγ (applied apically at pH 5.9) or AlexaFluor-488-labeled pIgA (applied basolaterally at pH 7.4) were incubated with FcRn-MDCK or pIgR-MDCK cells for a short pulse and then chased forthe indicated times. Fixed cells were prepared for immunofluorescence using an antibody against Rab7, Rab9 or LAMP2 and examined by confocal microscopy. Quantitative 3-D colocalization analyses are presented as Pearson's correlation coefficients. Values in histograms represent the mean and standard error from measurements taken from 11 to 19 cells percondition. Calculated p-values for the statistical significance of differences between pairwise combinations in the histograms are presented in Table S1. (A) Quantitative 3-D analysis of Fcγ/Rab7 and Fcγ/LAMP2 colocalizations as a function ofchase time in sub-volumes. Similar results were obtained for colocalization of Fcγ with Rab9 (data not shown). (B) Quantitative 3-D analysis of pIgA/Rab7 and pIgA/LAMP2 colocalizations as a function of chase time in sub-volumes. Similar results were obtained for colocalization of Fcγ with Rab9 (data not shown).

**Table S1.** Statistical significance of differences for pairwise combinations. p-values are presented for all pairwise combinations in the histograms shown in Figures 1–5, 7, S1 and S2. Tests of statistical significance were calculated using the two-tailed Student's *t*-test.
