# Supplementary material for: Comparison of FcRn- and pIgR-Mediated Transport in MDCK Cells by Fluorescence Confocal Microscopy
Source: Traffic. 2010 Jun 29;11(9):1205–20. doi: 10.1111/j.1600-0854.2010.01083.x (PMC2975666; doi:10.1111/j.1600-0854.2010.01083.x)
Supplement: Supplementary file 3 [file tra0011-1205-SD3.pdf]

Supplementary Table 1

Statistics for Histogram 1C
Fcγ / pIgA colocalization (whole cell volumes)
Significantly different ($p < 0.05$)

	2 min	5 min	10 min	20 min	30 min
**2 min**	-	0.0013	6.2E-08	1.1E-15	6.1E-12
**5 min**		-	1.9E-06	2.6E-16	5.4E-12
**10 min**			-	8.2E-11	1.4E-08
**20 min**				-	0.1569
**30 min**					-

Statistics for Histogram 1C
Fcγ / pIgA colocalization (sub-volumes)
Significantly different ($p < 0.05$)

		2 min			5 min			10 min			20 min			30 min		
		**Ap**	**MED**	**BL**	**Ap**	**MED**	**BL**	**Ap**	**MED**	**BL**	**Ap**	**MED**	**BL**	**Ap**	**MED**	**BL**
	**Ap**	-	0.5109	0.5157	0.1595	1.2E-04	0.2221	3.0E-07	9.2E-06	9.5E-09	1.9E-06	5.0E-10	1.3E-09	5.0E-05	1.9E-05	0.0003
**2 min**	**MED**		-	0.2283	0.4908	0.0009	0.3652	5.3E-07	1.2E-05	1.3E-08	2.7E-06	2.7E-10	2.0E-09	2.8E-05	2.5E-05	0.0003
	**BL**			-	0.0551	6.6E-05	0.1446	1.7E-07	6.5E-06	1.2E-08	1.5E-06	4.0E-10	3.3E-09	4.4E-05	1.6E-05	0.0002
	**Ap**				-	0.0027	0.5756	1.3E-06	1.7E-05	3.2E-08	3.3E-06	9.3E-10	2.3E-09	3.3E-05	3.0E-05	0.0004
**5 min**	**MED**					-	0.1917	4.9E-05	0.0002	6.0E-07	1.5E-05	3.4E-09	8.1E-09	9.6E-05	9.7E-05	0.0009
	**BL**						-	0.0001	9.0E-05	3.0E-06	2.9E-06	3.2E-09	2.7E-09	2.9E-05	2.0E-05	0.0003
	**Ap**							-	0.3684	0.0538	0.0035	5.7E-08	1.5E-07	0.0038	0.0075	0.0175
**10 min**	**MED**								-	0.5093	0.0409	1.7E-05	1.9E-05	0.0229	0.0615	0.0591
	**BL**									-	0.0788	5.2E-06	1.0E-05	0.0449	0.1182	0.1060
	**Ap**										-	0.0148	0.0145	0.6484	0.8607	0.7391
**20 min**	**MED**											-	0.9251	0.0727	0.0113	0.1258
	**BL**												-	0.0686	0.0109	0.1174
	**Ap**													-	0.5357	0.9578
**30 min**	**MED**														-	0.6369
	**BL**															-

Statistics for Histogram 1D
Fcγ fluorescence
Significantly different ($p < 0.05$)

		2 min			5 min			10 min			20 min			30 min		
		**Ap**	**MED**	**BL**	**Ap**	**MED**	**BL**	**Ap**	**MED**	**BL**	**Ap**	**MED**	**BL**	**Ap**	**MED**	**BL**
	**Ap**	-	1.6E-06	1.8E-05	0.0580	0.0010	4.4E-07	1.4E-06	2.9E-05	1.1E-06	4.0E-05	3.8E-06	1.1E-05	0.0006	0.0002	2.8E-06
**2 min**	**MED**		-	0.0032	0.0032	0.1048	0.0089	0.0312	0.0007	0.3233	0.0021	0.1213	0.1422	0.1706	0.0011	0.9460
	**BL**			-	0.0028	0.0092	0.0835	7.3E-07	3.3E-09	2.5E-06	0.0002	0.0007	0.0017	0.0068	0.0003	0.0046
	**Ap**				-	0.0247	0.0016	0.0167	0.0675	0.0079	0.0687	0.0130	0.0105	0.0182	0.1442	0.0038
**5 min**	**MED**					-	0.0108	0.7453	0.2142	0.2328	0.2897	0.4633	0.5273	0.8224	0.1173	0.1216
	**BL**						-	3.2E-05	7.2E-06	0.0004	5.2E-05	0.0007	0.0024	0.0121	0.0001	0.0117
	**Ap**							-	0.0038	0.0875	0.0419	0.4850	0.6187	0.9767	0.0141	0.0516
**10 min**	**MED**								-	2.8E-05	0.7807	0.0064	0.0216	0.1602	0.4189	0.0022
	**BL**									-	0.0029	0.3924	0.4110	0.3647	0.0025	0.4015
	**Ap**										-	0.0209	0.0414	0.2181	0.3666	0.0034
**20 min**	**MED**											-	0.9227	0.6671	0.0075	0.1600
	**BL**												-	0.7298	0.0146	0.1774
	**Ap**													-	0.0892	0.1865
**30 min**	**MED**														-	0.0016
	**BL**															-

Statistics for Histogram 1E
pIgA fluorescence
Significantly different ($p < 0.05$)

		2 min			5 min			10 min			20 min			30 min		
		**Ap**	**MED**	**BL**	**Ap**	**MED**	**BL**	**Ap**	**MED**	**BL**	**Ap**	**MED**	**BL**	**Ap**	**MED**	**BL**
	**Ap**	-	0.1475	2.6E-05	0.1527	8.1E-05	4.6E-05	0.0104	0.0010	0.0002	2.4E-05	0.0003	0.0220	2.9E-08	0.0030	0.1415
**2 min**	**MED**		-	5.3E-05	0.9308	0.0014	0.0002	0.1455	0.0224	0.0003	9.5E-05	0.0049	0.3484	4.3E-07	0.0717	0.6776
	**BL**			-	4.7E-05	0.0007	0.0067	4.7E-05	0.0003	0.0068	0.0074	0.0002	0.0001	0.0770	0.0001	0.0003
	**Ap**				-	0.0006	0.0001	0.1161	0.0121	0.0005	0.0001	0.0033	0.2882	1.1E-07	0.0532	0.7368
**5 min**	**MED**					-	0.0080	0.0125	0.0150	0.0213	0.0066	0.2392	0.0018	7.4E-06	0.0118	1.0E-04
	**BL**						-	0.0004	0.0018	0.8182	0.9872	0.0024	0.0003	0.0531	0.0006	0.0003
	**Ap**							-	0.3636	0.0015	0.000286	0.0898	0.4845	5.7E-07	0.7669	0.0447
**10 min**	**MED**								-	0.0036	0.001079	0.2055	0.0725	6.3E-07	0.5190	0.0002
	**BL**									-	0.809318	0.0086	0.0010	0.0600	0.0017	0.0005
	**Ap**										-	0.0019	0.0002	0.0528	0.0003	0.0001
**20 min**	**MED**											-	0.0163	1.9E-06	0.1183	0.0009
	**BL**												-	1.1E-07	0.2870	0.1103
	**Ap**													-	1.1E-07	1.1E-07
**30 min**	**MED**														-	0.0105
	**BL**															-

Supplementary Table 1 (cont)

Statistics for Histogram 2B
Fcγ / pIgA colocalization (whole cell volumes)
Significantly different ($p < 0.05$)

		5 min		10 min		30 min	
		BL	Ap	BL	Ap	BL	Ap
5 min	BL	-	0.6969	0.0007	0.0001	0.0019	0.0442
	Ap		-	0.0003	0.0005	0.0006	0.0199
10 min	BL			-	0.5240	0.6889	0.3989
	Ap				-	0.9956	0.4809
30 min	BL					-	0.4911
	Ap						-

Statistics for Histogram 2C
Fcγ / pIgA colocalization both applied to Basolateral
Significantly different ($p < 0.05$)

		5 min			10 min			30 min		
		Ap	MED	BL	Ap	MED	BL	Ap	MED	BL
	Ap	-	0.0004	6.7E-05	0.0029	0.0024	0.0039	0.0072	0.0088	0.0286
5 min	MED		-	0.0128	0.0113	0.2510	0.0169	0.1775	0.1073	0.0246
	BL			-	0.0002	0.0032	0.0002	0.0047	0.0020	0.0011
	Ap				-	0.1407	0.8039	0.2458	0.3772	0.8297
10 min	MED					-	0.1951	0.7763	0.5585	0.1453
	BL						-	0.3273	0.4920	0.6785
	Ap							-	0.7681	0.2361
30 min	MED								-	0.3449
	BL									-

Statistics for Histogram 2C
Fcγ / pIgA colocalization both applied to Apical
Significantly different ($p < 0.05$)

		5 min			10 min			30 min		
		Ap	MED	BL	Ap	MED	BL	Ap	MED	BL
	Ap	-	0.0021	0.0003	0.0067	0.0014	0.0003	0.0013	0.0008	0.0003
5 min	MED		-	0.0042	0.2129	0.1468	0.0057	0.4841	0.0380	0.0035
	BL			-	0.0002	3.9E-07	0.2283	0.0001	5.5E-06	0.1466
	Ap				-	0.0024	9.2E-05	0.0105	0.0006	0.0002
10 min	MED					-	6.1E-06	0.0904	0.0013	7.4E-07
	BL						-	8.8E-05	4.9E-05	0.0610
	Ap							-	0.0052	0.0003
30 min	MED								-	6.5E-06
	BL									-

Statistics for Histogram 2D
Fcγ fluorescence, applied to Basolateral
Significantly different ($p < 0.05$)

		5 min			10 min			30 min		
		Ap	MED	BL	Ap	MED	BL	Ap	MED	BL
	Ap	-	0.0117	5.0E-05	0.0295	0.0047	0.1947	0.0084	0.0035	0.0632
5 min	MED		-	0.0018	0.5550	0.0214	0.9895	0.1094	0.3349	0.3198
	BL			-	0.0063	0.0218	0.0445	0.0061	0.0005	0.0419
	Ap				-	0.0726	0.7139	0.3647	0.9049	0.5937
10 min	MED					-	0.1331	0.1755	0.0594	0.2478
	BL						-	0.3538	0.6537	0.4656
	Ap							-	0.2968	0.8395
30 min	MED								-	0.6111
	BL									-

Statistics for Histogram 2D
Fcγ fluorescence, applied to Apical
Significantly different ($p < 0.05$)

		5 min			10 min			30 min		
		Ap	MED	BL	Ap	MED	BL	Ap	MED	BL
	Ap	-	0.0399	0.0150	0.0359	0.1404	0.0074	0.0251	0.1003	0.0277
5 min	MED		-	0.0424	0.9708	0.1772	0.1362	0.5386	0.3325	0.5361
	BL			-	0.0392	0.0017	0.0538	0.0087	0.0208	0.0895
	Ap				-	0.1563	0.1327	0.5489	0.3041	0.5473
10 min	MED					-	0.0102	0.0261	0.6399	0.1036
	BL						-	0.1881	0.0313	0.4317
	Ap							-	0.0998	0.8631
30 min	MED								-	0.1656
	BL									-

Statistics for Histogram 2E
pIgA fluorescence, applied to Basolateral
Significantly different ($p < 0.05$)

		5 min			10 min			30 min		
		Ap	MED	BL	Ap	MED	BL	Ap	MED	BL
	Ap	-	0.0014	1.9E-06	0.1272	0.0015	0.0012	0.0010	0.0732	0.1005
5 min	MED		-	0.0006	0.0308	0.1137	0.0098	0.0044	0.0706	0.0010
	BL			-	0.0007	1.7E-05	0.8090	0.0439	0.0026	2.9E-06
	Ap				-	0.1512	0.0011	0.0007	0.5763	0.0341
10 min	MED					-	0.0053	0.0023	0.3455	0.0004
	BL						-	0.0509	0.0026	0.0007
	Ap							-	0.0008	0.0048
30 min	MED								-	0.0247
	BL									-

Statistics for Histogram 2E
pIgA fluorescence, applied to Apical
Significantly different ($p < 0.05$)

		5 min			10 min			30 min		
		Ap	MED	BL	Ap	MED	BL	Ap	MED	BL
	Ap	-	5.6E-06	3.2E-05	0.4506	5.6E-05	0.0005	0.5271	7.5E-06	0.0005
5 min	MED		-	0.0119	3.8E-06	0.1786	0.0073	3.6E-06	0.7943	0.0200
	BL			-	2.1E-05	0.0009	0.4836	2.1E-05	0.0219	0.3223
	Ap				-	3.9E-05	0.0004	0.1689	5.5E-06	0.0004
10 min	MED					-	0.0016	3.8E-05	0.1825	0.0013
	BL						-	0.0004	0.0360	0.6739
	Ap							-	5.1E-06	0.0004
30 min	MED								-	0.0324
	BL									-

Supplementary Table 1 (cont)

Statistics for Histogram 3B
Fcγ / EEA1 colocalization
Significantly different ($p < 0.05$)

		2 min			5 min			10 min			20 min			30 min		
		Ap	MED	BL	Ap	MED	BL	Ap	MED	BL	Ap	MED	BL	Ap	MED	BL
	Ap	-	9.1E-06	3.9E-15	4.5E-05	3.2E-05	7.1E-05	6.2E-07	3.7E-12	4.9E-16	6.7E-09	1.4E-11	1.4E-19	4.3E-08	1.0E-08	1.0E-18
2 min	MED		-	1.1E-08	0.7863	0.0201	0.0005	0.8134	0.0037	7.1E-08	0.0184	0.0028	7.7E-10	0.0859	0.0013	6.3E-10
	BL			-	6.2E-08	0.0008	0.7177	2.1E-09	1.4E-06	0.2537	1.5E-05	5.9E-06	0.2459	4.8E-07	0.0007	0.0240
	Ap				-	0.0118	0.0005	0.5862	0.0012	1.9E-07	0.0070	0.0008	4.7E-08	0.0353	0.0005	4.4E-07
5 min	MED					-	0.0069	0.0277	0.9860	0.0034	0.8282	0.8490	0.0003	0.2862	0.4089	0.0002
	BL						-	0.0006	0.0066	0.3291	0.0049	0.0087	0.8078	0.0020	0.0174	0.4487
	Ap							-	0.0044	1.8E-08	0.0253	0.0032	2.9E-11	0.1197	0.0015	8.6E-12
10 min	MED								-	2.6E-05	0.8087	0.7917	4.0E-09	0.1840	0.3106	1.6E-10
	BL									-	0.0002	0.0001	0.0189	4.5E-06	0.0066	0.0006
	Ap										-	0.6518	4.8E-07	0.3704	0.2781	7.5E-08
20 min	MED											-	4.0E-08	0.1324	0.4438	2.2E-09
	BL												-	1.7E-08	4.7E-05	0.2078
	Ap													-	0.0450	1.2E-08
30 min	MED														-	9.3E-06
	BL															-

Statistics for Histogram 3C
pIgA / EEA1 colocalization
Significantly different ($p < 0.05$)

		2 min			5 min			10 min			20 min			30 min		
		Ap	MED	BL	Ap	MED	BL	Ap	MED	BL	Ap	MED	BL	Ap	MED	BL
	Ap	-	0.2470	0.2891	0.0869	0.3186	0.9687	0.0001	0.0001	0.0003	0.0003	0.0006	0.0537	0.0008	6.4E-05	0.0599
2 min	MED		-	0.8989	0.8514	0.7762	0.2563	0.0043	0.0043	0.0647	0.0008	0.0013	0.7966	0.0015	0.0007	0.5205
	BL			-	0.9757	0.7080	0.2919	0.0030	0.0030	0.0461	0.0006	0.0011	0.9239	0.0013	0.0005	0.4358
	Ap				-	0.6690	0.0970	0.0006	0.0006	0.0052	0.0004	0.0013	0.9042	0.0015	0.0002	0.3225
5 min	MED					-	0.3245	0.0585	0.0585	0.3274	0.0138	0.0074	0.6419	0.0052	0.0160	0.8654
	BL						-	0.0001	0.0001	0.0003	0.0001	0.0006	0.0630	0.0008	6.8E-05	0.0632
	Ap							-	0.0351	0.0604	0.1768	0.0759	0.0006	0.0587	0.2268	0.0110
10 min	MED								-	0.0007	0.3520	0.9771	0.0001	0.6521	0.2605	0.0002
	BL									-	0.0056	0.0076	0.0029	0.0100	0.0049	0.1976
	Ap										-	0.4318	0.0005	0.2651	0.8435	0.0013
20 min	MED											-	0.0020	0.6555	0.3430	0.0019
	BL												-	0.0021	0.0002	0.2744
	Ap													-	0.2140	0.0028
30 min	MED														-	0.0013
	BL															-

Statistics for Histogram 3D
Fcγ and pIgA Colocalization with EEA1 (whole cell volumes)
Significantly different ($p < 0.05$)

		2 min		5 min		10 min		20 min		30 min	
		Fcγ/EEA1	pIgA/EEA1	Fcγ/EEA1	pIgA/EEA1	Fcγ/EEA1	pIgA/EEA1	Fcγ/EEA1	pIgA/EEA1	Fcγ/EEA1	pIgA/EEA1
2 min	Fcγ/EEA1	-	2.3E-14	3.1E-07	3.9E-07	1.3E-07	5.6E-07	2.0E-05	0.0005	0.0001	0.0026
	pIgA/EEA1		-	3.9E-07	0.0949	2.5E-12	9.6E-05	2.6E-07	0.0019	0.0002	0.0010
5 min	Fcγ/EEA1			-	1.3E-07	0.7854	0.0147	0.8410	0.2645	0.1870	0.5658
	pIgA/EEA1				-	8.9E-11	7.4E-05	2.5E-07	0.0009	0.0001	0.0004
10 min	Fcγ/EEA1					-	0.0340	0.9853	0.3662	0.2854	0.4742
	pIgA/EEA1						-	0.0708	0.4228	0.5397	0.0412
20 min	Fcγ/EEA1							-	0.4014	0.3228	0.5263
	pIgA/EEA1								-	0.8718	0.1806
30 min	Fcγ/EEA1									-	0.1375
	pIgA/EEA1										-

Statistics for Histogram 3E
EEA1 fluorescence
Significantly different ($p < 0.05$)

		2 min			5 min			10 min			20 min			30 min		
		Ap	MED	BL	Ap	MED	BL	Ap	MED	BL	Ap	MED	BL	Ap	MED	BL
	Ap	-	0.2422	0.0135	0.9019	0.1439	0.0042	0.7949	0.1735	0.0055	0.8573	0.2189	0.0137	0.7773	0.1388	0.0156
2 min	MED		-	0.0226	0.1743	0.8321	0.0090	0.2600	0.9724	0.0119	0.2076	0.8727	0.0230	0.1439	0.7017	0.0264
	BL			-	0.0082	0.0064	0.8247	0.0052	0.0034	0.6542	0.0024	0.0059	0.9259	0.0046	0.0225	0.6101
	Ap				-	0.0859	0.0022	0.6412	0.0999	0.0010	0.6987	0.1365	0.0084	0.8550	0.0923	0.0022
5 min	MED					-	0.0006	0.0734	0.6608	0.0013	0.0367	0.5627	0.0068	0.0311	0.7931	0.0020
	BL						-	0.0013	0.0002	0.7859	0.0001	0.0005	0.8562	0.0011	0.0093	0.8387
	Ap							-	0.0002	0.0006	0.8760	0.1457	0.0054	0.4588	0.1403	0.0013
10 min	MED								-	0.0007	0.0380	0.8200	0.0037	0.0505	0.5886	0.0002
	BL									-	0.0003	0.0010	0.6746	0.0005	0.0128	0.8976
	Ap										-	0.0795	0.0025	0.0005	0.1061	0.0001
20 min	MED											-	0.0062	0.0571	0.5246	0.0017
	BL												-	0.0048	0.0230	0.6336
	Ap													-	0.0798	0.0011
30 min	MED														-	0.0094
	BL															-

Supplementary Table 1 (cont)

Statistics for Histogram 4B
Fcγ / Rab11 colocalization
Significantly different ($p < 0.05$)

		2 min			5 min			10 min			20 min			30 min		
		**Ap**	**MED**	**BL**	**Ap**	**MED**	**BL**	**Ap**	**MED**	**BL**	**Ap**	**MED**	**BL**	**Ap**	**MED**	**BL**
	**Ap**	-	0.0002	1.4E-09	0.0487	0.4205	2E-06	0.6086	0.0670	0.0006	0.8726	0.0902	0.0093	0.9226	0.1421	0.0020
**2 min**	**MED**		-	5.8E-06	7.0E-06	0.0057	0.1049	0.0179	0.2457	0.1585	0.0009	0.0814	0.2912	0.0304	0.3165	0.0920
	**BL**			-	1.1E-09	3.7E-07	3.4E-05	0.0008	0.0006	0.0059	3.5E-05	0.0003	0.0364	0.0043	0.0107	0.0249
	**Ap**				-	0.0225	1.2E-07	0.5737	0.0071	5.2E-05	0.1046	0.0064	0.0020	0.2961	0.0292	0.0004
**5 min**	**MED**					-	0.0002	0.3533	0.2239	0.0015	0.3846	0.3358	0.0139	0.5496	0.2981	0.0020
	**BL**						-	0.0046	0.0357	0.8560	7.8E-05	0.0081	0.9202	0.0143	0.0898	0.5118
	**Ap**							-	0.0950	0.0039	0.6765	0.1338	0.0080	0.7254	0.1212	0.0032
**10 min**	**MED**								-	0.0421	0.0664	0.6941	0.0896	0.1480	0.9875	0.0265
	**BL**									-	0.0006	0.0137	0.9719	0.0087	0.0783	0.6772
	**Ap**										-	0.0856	0.0067	0.9997	0.1231	0.0013
**20 min**	**MED**											-	0.0442	0.2107	0.7432	0.0103
	**BL**												-	0.0134	0.1139	0.7040
	**Ap**													-	0.1870	0.0059
**30 min**	**MED**														-	0.0484
	**BL**															-

Statistics for Histogram 4C
pIgA / Rab11 colocalization
Significantly different ($p < 0.05$)

		2 min			5 min			10 min			20 min			30 min		
		**Ap**	**MED**	**BL**	**Ap**	**MED**	**BL**	**Ap**	**MED**	**BL**	**Ap**	**MED**	**BL**	**Ap**	**MED**	**BL**
	**Ap**	-	0.0385	0.0151	0.0142	0.0053	0.0039	0.0028	0.0028	0.0498	0.0006	0.0006	0.0078	3.1E-07	0.0228	0.2269
**2 min**	**MED**		-	0.7468	0.8509	0.5841	0.4336	0.0378	0.4369	0.3212	0.0030	0.1017	0.8863	1.8E-05	0.5689	0.0991
	**BL**			-	0.8815	0.8269	0.6278	0.0484	0.6438	0.1536	0.0035	0.1490	0.8130	1.5E-05	0.7423	0.0463
	**Ap**				-	0.6999	0.5142	0.0413	0.5220	0.1809	0.0031	0.1043	0.9431	1.8E-06	0.6577	0.0419
**5 min**	**MED**					-	0.7610	0.0529	0.7886	0.0603	0.0048	0.1554	0.5997	4.9E-07	0.8553	0.0130
	**BL**						-	0.0700	0.9524	0.0380	0.0059	0.2583	0.4093	7.7E-07	0.9805	0.0089
	**Ap**							-	0.0684	0.0125	0.1001	0.1891	0.0378	0.0051	0.0949	0.0068
**10 min**	**MED**								-	0.0215	0.0053	0.2041	0.4003	1.5E-07	0.9888	0.0029
	**BL**									-	0.0023	0.0030	0.1068	7.1E-08	0.1710	0.1597
	**Ap**										-	0.0123	0.0034	0.3063	0.0056	0.0009
**20 min**	**MED**											-	0.0517	5.4E-07	0.4201	0.0005
	**BL**												-	2.9E-08	0.6014	0.0134
	**Ap**													-	4.0E-05	1.4E-08
**30 min**	**MED**														-	0.0648
	**BL**															-

Statistics for Histogram 4D
Colocalization with Rab11 (whole cell volumes)
Significantly different ($p < 0.05$)

		2 min		5 min		10 min		20 min		30 min	
		**Fcγ/Rab11**	**pIgA/Rab11**	**Fcγ/Rab11**	**pIgA/Rab11**	**Fcγ/Rab11**	**pIgA/Rab11**	**Fcγ/Rab11**	**pIgA/Rab11**	**Fcγ/Rab11**	**pIgA/Rab11**
**2 min**	**Fcγ/Rab11**	-	6.1E-08	1.5E-05	3.8E-09	0.2067	5.4E-07	0.1386	0.01054	0.8151	0.1919
	**pIgA/Rab11**		-	7.7E-08	0.1844	0.0008	0.0469	0.0001	0.00363	0.0001	0.0001
**5 min**	**Fcγ/Rab11**			-	1.8E-08	0.0159	2.3E-08	0.0192	2.5E-06	0.0001	4.6E-06
	**pIgA/Rab11**				-	0.0005	0.1790	0.0001	0.00503	0.0001	1.3E-05
**10 min**	**Fcγ/Rab11**					-	0.0010	0.8572	0.00883	0.2074	0.0708
	**pIgA/Rab11**						-	0.0002	0.01446	0.0002	4.3E-05
**20 min**	**Fcγ/Rab11**							-	0.00470	0.1464	0.0428
	**pIgA/Rab11**								-	0.0468	0.0622
**30 min**	**Fcγ/Rab11**									-	0.5226
	**pIgA/Rab11**										-

Statistics for Histogram 4E
Rab11 fluorescence
Significantly different ($p < 0.05$)

		2 min			5 min			10 min			20 min			30 min		
		**Ap**	**MED**	**BL**	**Ap**	**MED**	**BL**	**Ap**	**MED**	**BL**	**Ap**	**MED**	**BL**	**Ap**	**MED**	**BL**
	**Ap**	-	0.0231	0.0019	0.1993	0.0056	0.0014	0.5402	0.0098	0.0005	0.2763	0.0074	0.0005	0.6658	0.0137	0.0003
**2 min**	**MED**		-	0.0060	0.0011	0.2520	0.0063	0.0082	0.5681	0.0042	0.0014	0.2930	0.0037	0.0043	0.7783	0.0042
	**BL**			-	5.4E-05	0.0084	0.0924	0.0012	0.0064	0.6654	2.1E-05	0.0007	0.6046	8.8E-05	2.3E-05	0.6960
	**Ap**				-	8.6E-05	0.0002	0.5262	0.0003	5.5E-06	0.6873	0.0002	1.2E-05	0.2539	0.0007	8.9E-06
**5 min**	**MED**					-	0.0066	0.0025	0.5110	0.0075	4.9E-05	0.7741	0.0061	0.0003	0.1791	0.0109
	**BL**						-	0.0010	0.0060	0.7445	7.2E-05	0.0005	0.7860	0.0002	5.3E-05	0.7877
	**Ap**							-	0.0039	0.0003	0.7073	0.0036	0.0003	0.7704	0.0061	0.0002
**10 min**	**MED**								-	0.0043	0.0002	0.6223	0.0036	0.0010	0.6357	0.0060
	**BL**									-	2.6E-06	0.0028	0.9585	1.2E-05	0.0009	0.9918
	**Ap**										-	4.1E-05	2.0E-06	0.3678	0.0001	4.8E-06
**20 min**	**MED**											-	0.0021	0.0005	0.1536	0.0048
	**BL**												-	1.0E-05	0.0007	0.9703
	**Ap**													-	0.0009	2.0E-05
**30 min**	**MED**														-	0.0017
	**BL**															-

Supplementary Table 1 (cont)

Statistics for Histogram 5B
Fcγ / Tf colocalization
Significantly different ($p < 0.05$)

		2 min			5 min			10 min			20 min			30 min		
		**Ap**	**MED**	**BL**	**Ap**	**MED**	**BL**	**Ap**	**MED**	**BL**	**Ap**	**MED**	**BL**	**Ap**	**MED**	**BL**
	**Ap**	-	0.2496	0.7050	0.0241	0.0015	0.0498	0.0074	0.0049	0.0216	3.1E-06	6.5E-09	7.3E-07	0.0142	0.0011	0.0169
**2 min**	**MED**		-	0.1288	0.0913	0.0050	0.1744	0.0131	0.0089	0.0598	1.2E-05	1.1E-08	1.9E-06	0.0271	0.0015	0.0045
	**BL**			-	0.0142	0.0013	0.0303	0.0059	0.0039	0.0146	1.8E-06	3.7E-09	3.8E-07	0.0112	0.0010	0.0024
	**Ap**				-	0.0866	0.8012	0.0606	0.0478	0.6028	0.0006	4.8E-06	0.0007	0.1719	0.0034	0.0162
**5 min**	**MED**					-	0.0635	0.4636	0.4470	0.2523	0.0525	0.0006	0.0854	0.9156	0.0165	0.1862
	**BL**						-	0.0488	0.0350	0.4683	0.0006	5.1E-06	0.0007	0.1289	0.0020	0.0112
	**Ap**							-	0.9823	0.1250	0.4314	0.0410	0.6211	0.4434	0.0797	0.6579
**10 min**	**MED**								-	0.1072	0.3811	0.0248	0.5690	0.4293	0.0649	0.6210
	**BL**									-	0.0052	0.0001	0.0087	0.3590	0.0048	0.0368
	**Ap**										-	0.0299	0.6077	0.0775	0.1615	0.7218
**20 min**	**MED**											-	0.0029	0.0044	0.9839	0.0538
	**BL**												-	0.1249	0.0881	0.9815
	**Ap**													-	0.0169	0.1931
**30 min**	**MED**														-	0.1260
	**BL**															-

Statistics for Histogram 5C
pIgA / Tf colocalization
Significantly different ($p < 0.05$)

		2 min			5 min			10 min			20 min			30 min		
		**Ap**	**MED**	**BL**	**Ap**	**MED**	**BL**	**Ap**	**MED**	**BL**	**Ap**	**MED**	**BL**	**Ap**	**MED**	**BL**
	**Ap**	-	1.6E-05	0.2674	0.0028	5.7E-05	0.8182	0.3730	0.4155	0.2406	0.0026	0.0171	0.0004	0.0073	0.0095	3.7E-05
**2 min**	**MED**		-	0.0001	0.0005	0.9635	0.0002	0.0028	0.0100	0.0001	1.4E-05	5.5E-07	6.9E-08	0.0002	1.1E-05	2.1E-08
	**BL**			-	0.0032	8.1E-05	0.4023	0.9387	0.1404	0.9554	0.0371	0.4394	0.0230	0.0319	0.1347	0.0035
	**Ap**				-	0.0042	0.0094	0.0239	0.1906	0.0024	0.0003	2.9E-05	2.1E-06	0.0010	0.0003	6.1E-07
**5 min**	**MED**					-	0.0002	0.0021	0.0093	0.0001	1.1E-05	2.9E-05	6.8E-06	5.0E-05	1.1E-05	1.5E-06
	**BL**						-	0.4757	0.3583	0.3901	0.0071	0.0734	0.0053	0.0097	0.0241	0.0005
	**Ap**							-	0.1884	0.9023	0.1231	0.6461	0.1137	0.0734	0.2871	0.0296
**10 min**	**MED**								-	0.1346	0.0085	0.0473	0.0074	0.0053	0.0168	0.0031
	**BL**									-	0.0242	0.3331	0.0145	0.0255	0.0951	0.0015
	**Ap**										-	0.0458	0.9121	0.5186	0.3904	0.1005
**20 min**	**MED**											-	0.0043	0.0544	0.2218	0.0005
	**BL**												-	0.5169	0.2643	0.0317
	**Ap**													-	0.2168	0.5825
**30 min**	**MED**														-	0.0234
	**BL**															-

Statistics for Histogram 5D
Colocalization with Tf (whole cell volumes)
Significantly different ($p < 0.05$)

		2 min		5 min		10 min		20 min		30 min	
		**Fcγ/Tf**	**pIgA/Tf**	**Fcγ/Tf**	**pIgA/Tf**	**Fcγ/Tf**	**pIgA/Tf**	**Fcγ/Tf**	**pIgA/Tf**	**Fcγ/Tf**	**pIgA/Tf**
**2 min**	**Fcγ/Tf**	-	0.0209	0.0001	5.5E-07	3.8E-06	0.0031	5.4E-09	0.0273	9.7E-11	0.3695
	**pIgA/Tf**		-	0.1073	0.0091	0.2231	0.9383	0.0219	0.0118	0.0050	0.0094
**5 min**	**Fcγ/Tf**			-	6.4E-06	0.0018	0.1414	2.7E-06	0.0098	1.0E-08	0.0571
	**pIgA/Tf**				-	0.0495	0.0141	0.5122	9.4E-06	0.6872	0.0001
**10 min**	**Fcγ/Tf**					-	0.2120	0.1699	0.0001	0.0275	0.0007
	**pIgA/Tf**						-	0.0273	0.0202	0.0072	0.0137
**20 min**	**Fcγ/Tf**							-	2.1E-07	0.3289	0.0001
	**pIgA/Tf**								-	3.8E-09	0.4953
**30 min**	**Fcγ/Tf**									-	0.0001
	**pIgA/Tf**										-

Statistics for Histogram 5E
Tf Fluorescence
Significantly different ($p < 0.05$)

		2 min			5 min			10 min			20 min			30 min		
		**Ap**	**MED**	**BL**	**Ap**	**MED**	**BL**	**Ap**	**MED**	**BL**	**Ap**	**MED**	**BL**	**Ap**	**MED**	**BL**
	**Ap**	-	0.0095	0.0008	0.7700	0.0017	0.0024	0.6921	0.0207	4.2E-05	0.8533	0.0544	0.0477	0.7629	0.0013	9.1E-06
**2 min**	**MED**		-	0.0039	0.0154	0.3079	0.0097	0.0142	0.9027	0.0011	0.0923	0.6436	0.1296	0.0223	0.2939	0.0017
	**BL**			-	0.0003	0.0114	0.6195	0.0008	0.0056	0.7783	0.0011	0.0305	0.7207	0.0006	0.0106	0.3294
	**Ap**				-	0.0039	0.0015	0.5733	0.0215	0.0001	0.7240	0.0447	0.0311	0.6175	0.0034	0.0003
**5 min**	**MED**					-	0.0284	0.0030	0.4158	0.0035	0.0303	0.8427	0.2335	0.0045	0.9993	0.0059
	**BL**						-	0.0025	0.0137	0.7501	0.0027	0.0539	0.9444	0.0016	0.0266	0.6893
	**Ap**							-	0.0327	0.0001	0.9976	0.0825	0.0507	0.9961	0.0024	2.1E-05
**10 min**	**MED**								-	0.0030	0.0819	0.7167	0.1430	0.0373	0.4106	0.0076
	**BL**									-	0.0016	0.0385	0.8168	0.0001	0.0029	0.3035
	**Ap**										-	0.0885	0.0450	0.9999	0.0382	0.0043
**20 min**	**MED**											-	0.2060	0.0730	0.8403	0.0647
	**BL**												-	0.0535	0.2318	0.9036
	**Ap**													-	0.0038	0.0001
**30 min**	**MED**														-	0.0047
	**BL**															-

Supplementary Table 1 (cont)

Statistics for Histogram 7B
Fcγ fluorescence
Significantly different ($p < 0.05$)

		- nocodazole			+ nocodazole		
		Ap	MED	BL	Ap	MED	BL
- nocodazole	Ap	-	8.7E-06	0.0031	0.0004	0.3032	0.0001
	MED		-	3.5E-07	0.0122	0.0021	8.8E-06
	BL			-	9.2E-05	0.1716	0.0055
+ nocodazole	Ap				-	0.0001	9.0E-06
	MED					-	0.0024
	BL						-

Statistics for Histogram 7B
pIgA fluorescence
Significantly different ($p < 0.05$)

		- nocodazole			+ nocodazole		
		Ap	MED	BL	Ap	MED	BL
- nocodazole	Ap	-	0.3124	0.0023	0.0001	0.0050	0.0007
	MED		-	0.0047	2.8E-05	0.0066	0.0002
	BL			-	0.0032	0.5060	2.9E-05
+ nocodazole	Ap				-	0.0018	0.0003
	MED					-	0.0008
	BL						-

Statistics for Supplementary Histogram 1A
Colocalization with EEA1 (whole cell volumes)
Significantly different ($p < 0.05$)

		Fcγ / EEA1		pIgA / EEA1		Fcγ / pIgA / EEA1	
		5 min chase	20 min chase	5 min chase	20 min chase	5 min chase	20 min chase
Fcγ / EEA1	5 min chase	-	0.8448	0.0009	0.5126	0.0006	0.0433
	20 min chase		-	0.0082	0.7686	0.0061	0.1452
pIgA / EEA1	5 min chase			-	0.0005	0.5281	0.0008
	20 min chase				-	0.0006	0.0657
Fcγ / pIgA / EEA1	5 min chase					-	0.0003
	20 min chase						-

Statistics for Supplementary HIstogram 1C
Colocalization
Significantly different ($p < 0.05$)

			Fcγ / EEA1					
			Ap		MED		BL	
			5 min chase	20 min chase	5 min chase	20 min chase	5 min chase	20 min chase
Fcγ / EEA1	Ap	5 min chase	-	0.0804	0.5881	0.3765	0.0093	0.2059
		20 min chase		-	0.0300	0.4503	0.0012	0.0101
	MED	5 min chase			-	0.1854	0.0079	0.3592
		20 min chase				-	0.0081	0.0715
	BL	5 min chase					-	0.1202
		20 min chase						-
pIgA / EEA1	Ap	5 min chase						
		20 min chase						
	MED	5 min chase						
		20 min chase						
	BL	5 min chase						
		20 min chase						
Fcγ / pIgA	Ap	5 min chase						
		20 min chase						
	MED	5 min chase						
		20 min chase						
	BL	5 min chase						
		20 min chase						
Fcγ / pIgA / EEA1	Ap	5 min chase						
		20 min chase						
	MED	5 min chase						
		20 min chase						
	BL	5 min chase						
		20 min chase						

			pIgA / EEA1					
			Ap		MED		BL	
			5 min chase	20 min chase	5 min chase	20 min chase	5 min chase	20 min chase
Fcγ / EEA1	Ap	5 min chase	0.0045	0.2333	0.0063	0.1007	0.0042	0.4749
		20 min chase	0.0016	0.7782	0.0011	0.8674	0.0016	0.0413
	MED	5 min chase	0.0029	0.1209	0.0031	0.0503	0.0027	0.7246
		20 min chase	0.0075	0.6958	0.0062	0.4127	0.0073	0.1762
	BL	5 min chase	0.3596	0.0100	0.5900	0.0046	0.3360	0.1243
		20 min chase	0.0438	0.0513	0.0637	0.0188	0.0415	0.7225
pIgA / EEA1	Ap	5 min chase	-	0.0080	0.5481	0.0040	0.9575	0.0702
		20 min chase		-	0.0095	0.6895	0.0078	0.1118
	MED	5 min chase			-	0.0047	0.4979	0.0895
		20 min chase				-	0.0039	0.0501
	BL	5 min chase					-	0.0682
		20 min chase						-
Fcγ / pIgA	Ap	5 min chase						
		20 min chase						
	MED	5 min chase						
		20 min chase						
	BL	5 min chase						
		20 min chase						
Fcγ / pIgA / EEA1	Ap	5 min chase						
		20 min chase						
	MED	5 min chase						
		20 min chase						
	BL	5 min chase						
		20 min chase						

			Fcγ / pIgA					
			Ap		MED		BL	
			5 min chase	20 min chase	5 min chase	20 min chase	5 min chase	20 min chase
Fcγ / EEA1	Ap	5 min chase	0.0313	0.0351	0.0838	0.0617	0.0120	0.5678
		20 min chase	0.0027	0.7298	0.0065	0.7211	0.0012	0.1503
	MED	5 min chase	0.0378	0.0102	0.1252	0.0258	0.0096	0.2162
		20 min chase	0.0227	0.2835	0.0411	0.3110	0.0113	0.6184
	BL	5 min chase	0.2456	0.0003	0.0425	0.0017	0.7918	0.0011
		20 min chase	0.3878	0.0040	0.8013	0.0105	0.1474	0.0650
pIgA / EEA1	Ap	5 min chase	0.0321	0.0004	0.0019	0.0024	0.1690	0.0008
		20 min chase	0.0195	0.5667	0.0326	0.5692	0.0107	0.3807
	MED	5 min chase	0.0627	0.0005	0.0039	0.0028	0.3429	0.0011
		20 min chase	0.0085	0.9017	0.0137	0.8718	0.0048	0.1736
	BL	5 min chase	0.0277	0.0008	0.0021	0.0023	0.1549	0.0007
		20 min chase	0.3081	0.0225	0.5551	0.0315	0.1501	0.0650
Fcγ / pIgA	Ap	5 min chase	-	0.0011	0.3071	0.0048	0.3098	0.0041
		20 min chase		-	0.0018	0.9536	0.0005	0.0660
	MED	5 min chase			-	0.0076	0.0424	0.0126
		20 min chase				-	0.0027	0.1093
	BL	5 min chase					-	0.0014
		20 min chase						-
Fcγ / pIgA / EEA1	Ap	5 min chase						
		20 min chase						
	MED	5 min chase						
		20 min chase						
	BL	5 min chase						
		20 min chase						

			Fcγ / pIgA / EEA1					
			Ap		MED		BL	
			5 min chase	20 min chase	5 min chase	20 min chase	5 min chase	20 min chase
Fcγ / EEA1	Ap	5 min chase	0.0081	0.7811	0.0075	0.5886	0.0028	0.0128
		20 min chase	0.0013	0.1587	0.0012	0.3622	0.0006	0.0015
	MED	5 min chase	0.0045	0.4425	0.0056	0.3567	0.0016	0.0119
		20 min chase	0.0108	0.5472	0.0103	0.8178	0.0052	0.0102
	BL	5 min chase	0.8581	0.0136	0.8047	0.0283	0.3064	0.7152
		20 min chase	0.0879	0.1632	0.0813	0.1586	0.0355	0.1719
pIgA / EEA1	Ap	5 min chase	0.2427	0.0098	0.2642	0.0196	0.7225	0.1730
		20 min chase	0.0110	0.3462	0.0106	0.5662	0.0054	0.0120
	MED	5 min chase	0.5833	0.0088	0.6395	0.0237	0.4613	0.3203
		20 min chase	0.0054	0.1702	0.0052	0.3347	0.0025	0.0054
	BL	5 min chase	0.2057	0.0094	0.2190	0.0192	0.7395	0.1566
		20 min chase	0.1114	0.3716	0.1062	0.2993	0.0584	0.1627
Fcγ / pIgA	Ap	5 min chase	0.1146	0.0367	0.0974	0.0586	0.0360	0.3998
		20 min chase	0.0005	0.0806	0.0005	0.2323	0.0002	0.0006
	MED	5 min chase	0.0077	0.0851	0.0072	0.1024	0.0039	0.0711
		20 min chase	0.0032	0.1104	0.0031	0.2536	0.0014	0.0030
	BL	5 min chase	0.5822	0.0147	0.5257	0.0310	0.1715	0.8982
		20 min chase	0.0014	0.8344	0.0013	0.8643	0.0003	0.0015
Fcγ / pIgA / EEA1	Ap	5 min chase	-	0.0111	0.9171	0.0279	0.2690	0.5222
		20 min chase		-	0.0105	0.7617	0.0054	0.0179
	MED	5 min chase			-	0.0268	0.2889	0.4757
		20 min chase				-	0.0147	0.0348
	BL	5 min chase					-	0.1631
		20 min chase						-

Statistics for Supplementary Histogram 2A
Fcγ / Rab7 colocalization
Significantly different ($p < 0.05$)

		Ap		MED		BL	
		10 min chase	30 min chase	10 min chase	30 min chase	10 min chase	30 min chase
Ap	10 min chase	-	0.5834	0.0948	0.3170	0.0214	0.2679
	30 min chase		-	0.1009	0.2057	0.0543	0.1745
MED	10 min chase			-	0.5160	0.0699	0.6794
	30 min chase				-	0.1295	0.8700
BL	10 min chase					-	0.2014
	30 min chase						-

Statistics for Supplementary Histogram 2A
Fcγ / LAMP2 colocalization
Significantly different ($p < 0.05$)

		Ap		MED		BL	
		10 min chase	30 min chase	10 min chase	30 min chase	10 min chase	30 min chase
Ap	10 min chase	-	0.3123	0.5820	0.1018	0.0081	0.0081
	30 min chase		-	0.2414	0.6321	0.0022	0.0022
MED	10 min chase			-	0.1367	0.2070	0.2070
	30 min chase				-	0.0002	0.1367
BL	10 min chase					-	0.0002
	30 min chase						-

Statistics for Supplementary Histogram 2B
pIgA / Rab7 colocalization
Significantly different ($p < 0.05$)

		Ap		MED		BL	
		10 min chase	30 min chase	10 min chase	30 min chase	10 min chase	30 min chase
Ap	10 min chase	-	0.8360	0.1597	0.0420	0.0417	0.2378
	30 min chase		-	0.1217	0.0210	0.0242	0.2507
MED	10 min chase			-	0.0795	0.0624	0.8427
	30 min chase				-	0.8703	0.5615
BL	10 min chase					-	0.5956
	30 min chase						-

Statistics for Supplementary Histogram 2B
pIgA / LAMP2 colocalization
Significantly different ($p < 0.05$)

		Ap		MED		BL	
		10 min chase	30 min chase	10 min chase	30 min chase	10 min chase	30 min chase
Ap	10 min chase	-	0.0160	0.6591	0.0997	0.3893	0.7661
	30 min chase		-	0.0003	0.3454	0.0002	0.0005
MED	10 min chase			-	0.0257	0.4200	0.8087
	30 min chase				-	0.0163	0.0331
BL	10 min chase					-	0.3074
	30 min chase						-
